# *Leishmania donovani* Targets Dicer1 to Downregulate miR-122, Lower Serum Cholesterol, and Facilitate Murine Liver Infection

**DOI:** 10.1016/j.chom.2013.02.005

**Published:** 2013-03-13

**Authors:** June Ghosh, Mainak Bose, Syamal Roy, Suvendra N. Bhattacharyya

**Affiliations:** 1 RNA Biology Research Laboratory, Molecular and Human Genetics Division, CSIR-Indian Institute of Chemical Biology, Kolkata 700032, India; 2 Infectious Diseases and Immunology Division, CSIR-Indian Institute of Chemical Biology, Kolkata 700032, India

## Abstract

*Leishmania donovani* causes visceral leishmaniasis (VL) where the parasite infects and resides inside liver and spleen tissue macrophages. Given the abnormal lipid profile observed in VL patients, we examined the status of serum lipids in an experimental murine model of VL. The murine VL liver displayed altered expression of lipid metabolic genes, many of which are direct or indirect targets of the liver-specific microRNA-122. Concomitant reduction of miR-122 expression was observed in VL liver. High serum cholesterol caused resistance to *L. donovani* infection, while downregulation of miR-122 is coupled with low serum cholesterol in VL mice. Exosomes secreted by the infective parasites caused reduction in miR-122 activity in hepatic cells. *Leishmania* surface glycoprotein gp63, a Zn-metalloprotease, targets pre-miRNA processor Dicer1 to prevent miRNP formation in *L. donovani*-interacting hepatic cells. Conversely, restoration of miR-122 or Dicer1 levels in VL mouse liver increased serum cholesterol and reduced liver parasite burden.

## Graphical abstract

**Graphical Abstract:**

**Figure:**
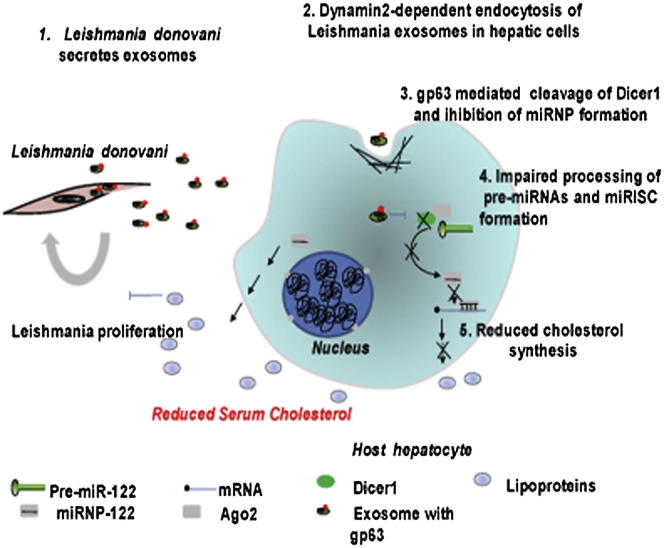

**Highlights:**

► *Leishmania* infection reduces liver miR-122 and lowers serum cholesterol ► *Leishmania* metalloprotease gp63 is required for inhibition of hepatic miR-122 activity ► gp63 cleaves DICER1 to downregulate miRNP-122 formation in hepatocytes ► Restoration of miR-122 elevates serum cholesterol to reduce liver parasite load

## Introduction

Visceral leishmaniasis (VL) is caused by the protozoan parasite *Leishmania donovani* or *Leishmania infantum* and is the most fatal form of this parasitic disorder (Murray et al., 2005). The parasite infects the spleen and liver of infected individuals and resides within the macrophages to escape host immune response (Olivier et al., 2005) It shows a dimorphic life cycle, residing as flagellate promastigotes in the midgut of the sand fly vector and as aflagellate amastigotes in the mammalian host (Desjardins and Descoteaux, 1998; Engwerda et al., 2004). Liver is the primary organ that gets infected in the early phase of infection where the parasites survive within the tissue macrophage Küpffer cells, while the liver parenchyma remains noninfected (Beattie et al., 2010).

VL patients show hypolipidemia characterized by reduced serum total cholesterol and lipoproteins (Lal et al., 2007). Interestingly, hyperlipidemia is associated with resistance to VL (Ghosh et al., 2012). In experimental VL, reduced membrane cholesterol in infected macrophages leads to increased membrane fluidity affecting its antigen-presenting ability (Chakraborty et al., 2005). Liposomal formulation of cholesterol is known to offer protection in infected hamsters (Banerjee et al., 2009). Although the involvement of cholesterol in controlling VL is evident from these studies, little is known about the influence of *Leishmania* on host lipid metabolism.

MicroRNAs (miRNAs), the 20–22 nt long posttranscriptional regulators, mediate gene repression primarily by inducing translational repression or degradation of target mRNAs to affect almost all physiological processes including metabolic processes in higher eukaryotes (Filipowicz et al., 2008; Krützfeldt and Stoffel, 2006). Precursors to miRNAs, primary miRNA transcripts (pri-miRNAs) are processed by microprocessor Drosha-DGCR8 in the nucleus to generate precursor miRNAs (pre-miRNAs), which are subsequently processed to the mature form by RNase III endonuclease Dicer1 in the cytoplasm (Filipowicz et al., 2008). The miRNA encoding strand of miRNA duplex gets loaded to Argonaute proteins by DICER1 and TAR RNA-binding proteins (TRBPs) to form active microRNA ribonucleoprotein complexes (miRNPs). miR-122, a miRNA expressed abundantly in liver, modulates a wide range of liver functions. miR-122 comprises more than 70% of the liver miRNA pool and is largely responsible for liver homeostasis and lipid metabolism (Chang et al., 2004; Girard et al., 2008). Antisense oligonucleotides against miR-122 confirmed its role in fatty acid and cholesterol metabolism (Elmén et al., 2008; Esau et al., 2006). Therefore, it is an interesting possibility that parasite infection controls liver miR-122 in order to modulate serum cholesterol.

*L. donovani* interacts with its target cell either by cell-cell contact or by secreting exosomes containing virulence factors (Silverman et al., 2010). The surface metalloprotease gp63, a membrane-bound glycosylphosphatidylinositol (GPI)-anchored glycoprotein of 63 kDa, is a known virulence factor present in *Leishmania* exosomes that serves as a ligand for the macrophage complement receptor (Brittingham et al., 1995). This *Leishmania* surface protease cleaves multiple intracellular proteins and participates in p38 mitogen-activated protein (MAP) kinase inactivation (Hallé et al., 2009). gp63 is also responsible for selective degradation of eIF4E in *L. donovani*-infected macrophages (Jaramillo et al., 2011).

We have shown in this study that *L. donovani* infection downregulates miR-122 and genes involved in cholesterol biosynthesis in infected mouse livers to reduce serum cholesterol. We have also found that restoration of miR-122 induces revival of serum cholesterol and reduction in liver parasite count. *Leishmania* metalloprotease gp63, a component of *Leishmania* exosomes, gets internalized to degrade Dicer1 in hepatic cells. It inhibits Dicer1-mediated pre-miR-122 processing to restrict miRNP formation and prevents miR-122 activity in *L. donovani*-interacting hepatocytes.

## Results

### *L. donovani* Alters Serum Lipid Profiles of Infected Animals

**Figure 1:**
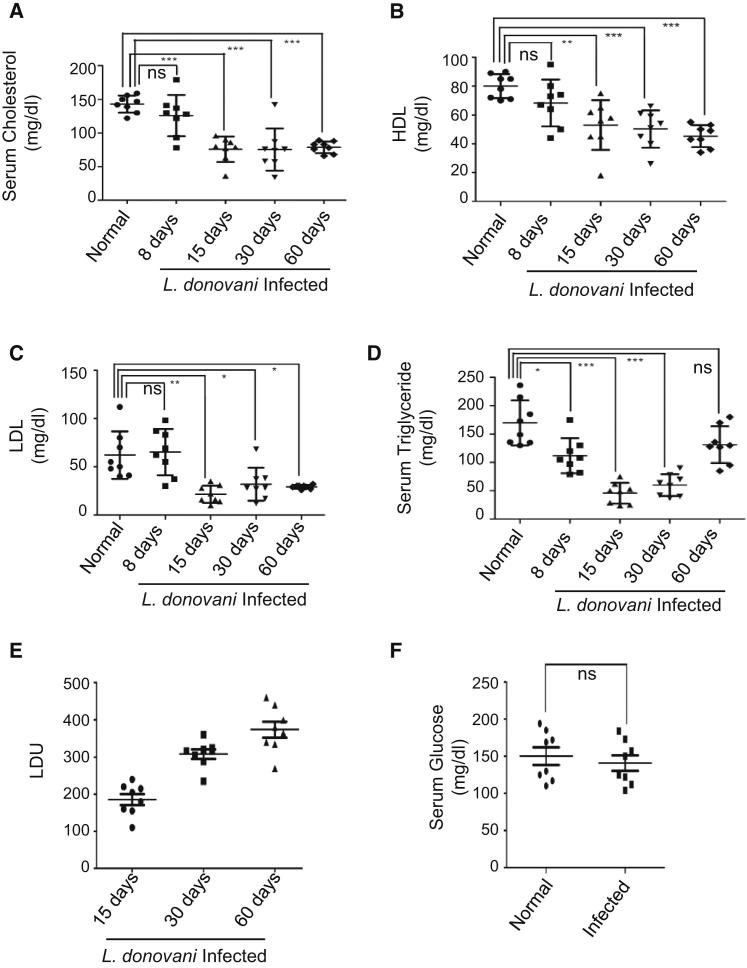
Reduction in Serum Lipids with Progress of *L. donovani* Infection in Mouse Liver (A) Level of total serum cholesterol in *L. donovani*-infected BALB/c mice. Serum cholesterol levels in mice infected for 8, 15, 30, or 60 days were measured and plotted individually. (B–D) Levels of HDL (B), LDL (C), and triglyceride (D) in the blood serum of 8, 15, 30, or 60 days of infected or age-matched normal animals. (E) Liver parasite load in individual animals from different experimental groups estimated and plotted as LDU against infection time. (F) Level of serum glucose in *L. donovani*-infected (1 or 2 months) and normal animals. Data represent mean ± SEM. For each experiment n = 8. Significance levels: ns (not significant), p > 0.05, ^∗^p < 0.05, ^∗∗^p < 0.001, ^∗∗∗^p < 0.0001. See also Table S1 and Figure S1.

Abnormal lipid profiles identified in VL patients (Ghosh et al., 2011; Liberopoulos et al., 2002) made us interested to look at the status of serum lipids in *L. donovani*-infected mice. Measurement of total cholesterol showed a gradual lowering in serum cholesterol with almost 50% reduction at 60 days postinfection (p.i.) in *L.donovani*-infected BALB/c mice. Major serum lipoproteins like high-density lipoprotein (HDL) and low-density lipoprotein (LDL) also showed similar trends (Figures 1A–1C). Serum triglyceride dropped up to 65% until 30 days p.i. with a partial recovery at 60 days p.i. (Figure 1D). An inverse correlation between serum cholesterol level and parasite load was evident (Figure 1E and Table S1). Histological examination of the infected liver section also revealed a gradual increase in both number and size of granuloma, with progress of infection where parasites were also visible (Figures S1A and S1B). Overall, we observed a substantial difference in serum lipid profiles in *L.donovani*-infected animals, whereas serum glucose level remained unaffected (Figure 1F). Similar changes in serum cholesterol were also documented in *L.donovani* amastigote-infected mouse livers (Figures S1C and S1D).

### Altered Expression of Lipid Metabolic Genes in *Leishmania*-Infected Mouse Liver

**Figure 2:**
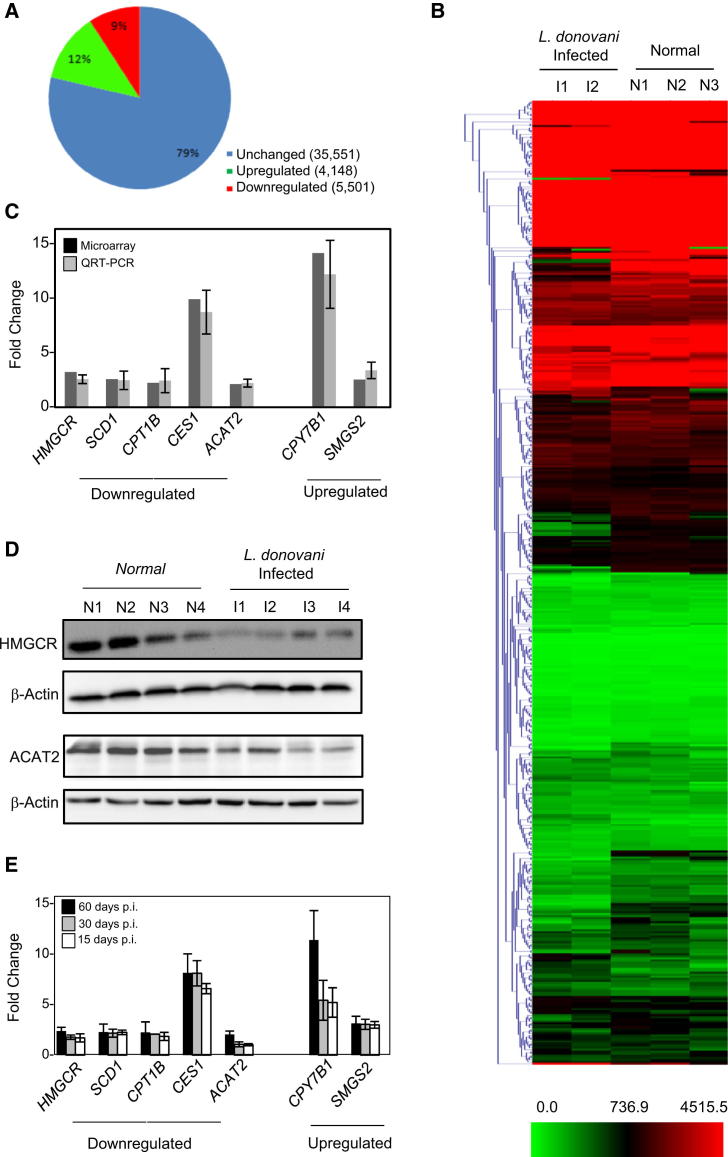
Altered Expression of Lipid Metabolizing Genes in the Livers of *L. donovani*-Infected Animals (A–C) Whole-genome microarray analysis revealed differentially expressed genes in *L.donovani*-infected liver. A pie chart representation of number of genes that showed altered expression in mouse livers after 60 days p.i. is shown. RNA was isolated from normal (n = 3) or infected (n = 2) livers, and whole genome microarray analysis was done (A). Heatmap of lipid metabolic genes (B). Fold changes obtained from both real-time quantification and microarray analysis were plotted for the key lipid metabolizing genes that showed either an increased or decreased expression in infected livers (C). (D) Decreased expression of cholesterol anabolic proteins HMGCR or ACAT2 in infected mouse liver. Liver tissue extracts were western blotted for HMGCR or ACAT2 detection. β-actin was used as loading control. n = 4 for noninfected control and 60 days p.i. (E) Changes in expression of important lipid metabolic genes in infected animal livers with progress of infection. Respective fold changes in expression were quantified by real-time PCR. For all real-time estimations, n ≥ 3. SD was from three independent measurements. Data represented as ±SEM. See also Table S2.

In order to dissect the cause of these abnormalities in the lipid profile of *L. donovani*-infected animals, we checked the expression levels of lipid metabolic genes in infected mouse liver. A whole-genome microarray analysis was done using an Illumina Mouse WG-6 v2 BeadChip gene expression array. Of 45,200 total transcripts analyzed, 9,659 showed a differential expression (5,501 were downregulated, and 4,148 were upregulated) with more than or equal to 2-fold changes in their expression in infected livers (Figure 2A). The whole microarray data have been uploaded to the Gene Expression Omnibus database (accession number GSE38985).

Scrutiny of differentially expressed gene clusters revealed that a large number of genes showing reduced expression in infected livers are related to lipid metabolism (83 showed more than or equal to 2-fold changes among 476 genes considered). A heatmap was generated showing differences in expression of lipid metabolizing genes between normal (n = 3) and infected (n = 2) groups (Figure 2B). A list comprising lipid metabolizing genes with fold change (upregulated or downregulated) greater than or equal to two are provided as a table (Table S2). In summary, the gene expression data suggest that a major alteration in lipid metabolism occurs in *L. donovani*-infected mouse liver. Among the genes listed, *HMGCR* (hydroxyl-3-methylglutaryl coenzyme A reductase), the rate-limiting enzyme for de novo cholesterol biosynthesis (fold downregulation = 2.9), and *CYP7B1* (25-hydroxycholesterol-7alpha-hydroxylase), a member of the monooxygenase cytochrome P450 superfamily that catalyzes the first step of catabolism of cholesterol to bile acid (fold upregulation = 12.71), are of key importance in cholesterol metabolism. The validation of the microarray data was performed by real-time quantification of seven (five showed downregulation and two showed upregulation in microarray analysis) of these genes. All of them showed proportional changes in their expression in infected mouse livers (Figure 2C). Changes in protein levels for HMGCR and ACAT2 were also confirmed by western blot analysis (Figure 2D). Interestingly, these genes showed altered expression even during the early stage of infection when the serum cholesterol level also starts to decrease (Figure 2E). The microarray data suggested an overall downregulation in cholesterol-synthesizing genes and selective upregulation of cholesterol catabolic genes that result in the lowering of serum cholesterol in *Leishmania*-infected mice.

### *Leishmania* Infection Reduces miR-122 Levels in Mouse Liver

**Figure 3:**
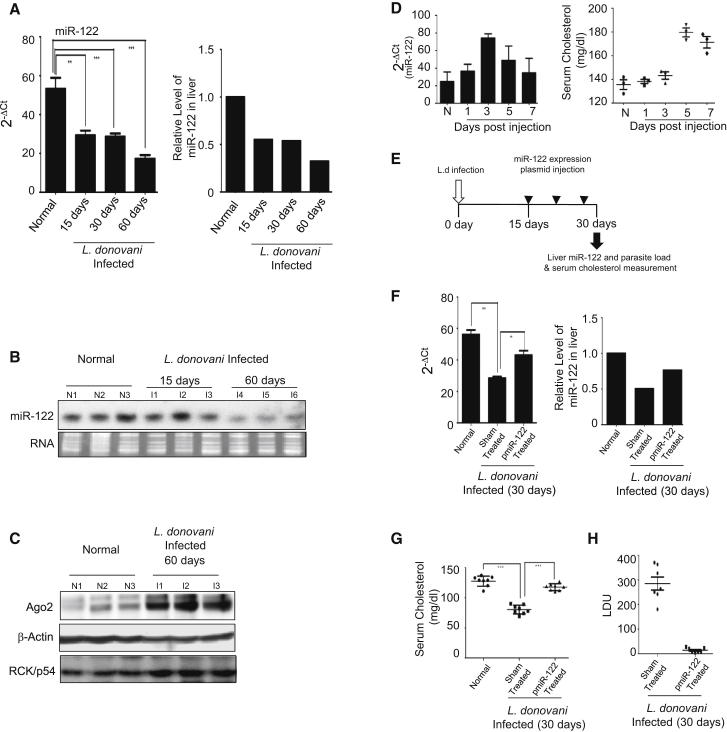
miR-122 Acts as a Modulator of Parasitic Infection in *L. donovani*-Infected Mouse Liver (A and B) Downregulation of miR-122 in *L. donovani*-infected mouse liver. Liver miR-122 levels in *L. donovani*-infected BALB/c mice were quantified by real-time quantification. U6 small nuclear RNA (snRNA) was used for normalization. miR-122 content in age-matched noninfected animals were used as reference value (A). Northern blot to detect miR-122 changes in infected mouse livers (B). Ethidium bromide (EtBr)-stained gel was used for loading control. For real-time quantification n = 8 for each time point. (C) Levels of AGO2 and RCK/p54 protein in *L. donovani*-infected mice. Western blot analysis for AGO2 and RCK/p54 proteins in liver extracts from normal and infected animals. β-actin was used as loading control. (D) Changes in expression of miR-122 and the serum cholesterol level in mice after introduction of miR-122 expression plasmid pmir122 through tail vein injection. Animals were sacrificed after indicated time; liver miR-122 and serum cholesterol levels were measured. (E) A schematic representation of the time course of exogenous miR-122 expression in infected mouse liver. Infection time is marked by a white arrow, and the black arrow defines the time of sacrifice 30 days p.i. The black arrowheads mark the injection time of miR-122 expression plasmids. (F–H) Effect of upregulation of miR-122 on infection level in mouse livers. Infected animals were treated as per the schedule described in (E) and were sacrificed after a month. Relative miR-122 expression (F), serum cholesterol level (G), and hepatic parasite load (H) were compared in pmiR-122-treated versus sham-treated groups. Data represent mean ± SEM. For all experiments, n = 8. ^∗∗∗^p < 0.0001, ^∗∗^p < 0.001. L.d, *L.donovani*. See also Table S3 and Figure S2.

A more detailed analysis of the microarray data highlighted an interesting finding: several lipid-metabolizing genes showing differential expression in the livers of infected animals are direct or indirect targets of miR-122 (Table S3). Among them, *HMGCR*, a rate-determining enzyme in cholesterol biosynthesis (fold downregulation = 2.9), and *FASN* (fatty acid synthase), controlling fatty acid synthesis (fold downregulation = 2.7), are known to be reciprocally regulated by miR-122 (Esau et al., 2006). Connection between miR-122 and lipid metabolism is well documented in mammals (Girard et al., 2008). Therefore, we may expect reduced miR-122 activity in *L. donovani*-infected livers. Levels of miR-122 in the total RNA isolated from the livers of normal and infected animals were determined. Real-time quantification revealed a gradual downregulation of miR-122 in mouse livers with progressive infection (Figure 3A). Northern blot analysis corroborated this finding (Figure 3B). A similar decrease in the miR-122 level was also observed in animals infected with *L.donovani* amastigotes (Figure S2A). Reduced miR-122 activity could not be due to lower expression of miRNP components, as we did not find any reduction in miRNP-interacting protein RCK/p54 in infected livers (Figure 3C). Interestingly, AGO2, the key miRNP component, showed an increased expression in infected liver. Therefore, *L. donovani* infection reduces liver miR-122 to control the serum cholesterol level.

### Exogenous Expression of miR-122 Can Rescue Serum Cholesterol and Clear Hepatic Parasite Load

In order to understand the significance of miR-122 downregulation during the *L. donovani* infection process, we aimed to complement the reduced miR-122 level in the livers of infected animals. To that end, we injected pmir122, a miR-122 expression plasmid with a constitutive U6 promoter, in noninfected mouse via tail vein and monitored miR-122 levels in its liver until 7 days postinjection. The miR-122 level was increased and was at a maximum after 3 days, whereas the serum cholesterol level started to show changes at 3 days and reached a maximum at 5 days p.i.(Figure 3D). To highlight the protective role of miR-122, we injected miR-122 expression plasmid in animals after 15 days p.i. when initial infection was already established. Animals received three doses of pmir122 injection, and the parasite load was monitored at 30 days p.i. (Figure 3E). We documented an increase in miR-122 level with a concomitant elevation in serum cholesterol (Figures 3F and 3G). This also induced reversal of expression of key cholesterol metabolic genes (data not shown). Interestingly, the hepatic parasite load was reduced significantly compared to sham-treated control animals (Figure 3H) showing the protective action of miR-122 against *L. donovani* infection.

### *L. donovani* Interacts with Human Hepatocytes and Impairs miRNA-Mediated Repression

**Figure 4:**
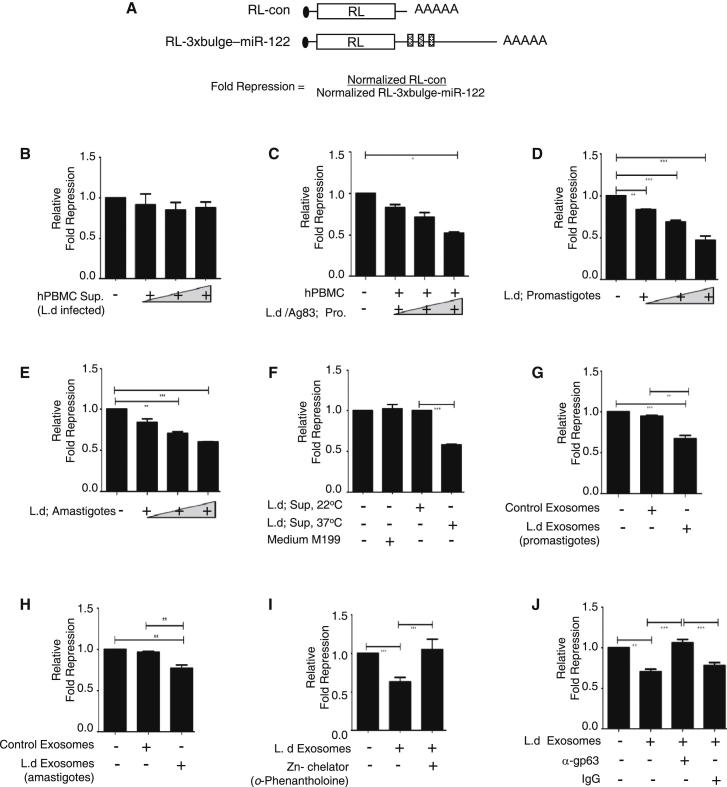
Inhibition of miR-122 in Huh7 Cells by *L. donovani* (A) Schematic picture of the miR-122 reporter used to measure the miR-122 activity in Huh7 cells. For relative fold repression, the calculation level of repression for controls was always settled to 1. (B–E) Modulation of miR-122 activity in Huh7 cells by *L.donovani*. Huh7 expressing either the RL-con or RL-3 × bulge-miR-122 *Renilla* luciferase reporter along with firefly luciferase encoding control mRNA were grown and treated differently (as described below) before the luciferase activities were measured and relative fold repression values calculated. (B) Huh7 cultured with *Leishmania*-infected human PBMC supernatant. (C) Huh7 cocultured with human PBMC and with increasing numbers of *L. donovani* parasite. Huh7 cultured with *L. donovani* promastigotes (1:1, 1:10, 1:100) (D) or amastigotes (1:1, 1:10, 1:100) (E). (F–J) Exosomes released by *L.donovani* affects miR-122 activity in Huh7 cells. As described above, Huh7 cells expressing miR-122 reporter or control mRNAs were treated with *L.donovani* culture supernatant (grown at either 22°C or 37°C) or control medium used for parasite growth (M199), and fold repression values were calculated (F). Exosomes isolated from the *L. donovani* promastigote-grown (G) or amastigote-grown (H) medium or control medium were also used to test its effect on miR-122-mediated repression. In similar experiments, *L.donovani* exosomes either pretreated with the Zn-chelator *o*-Phenantholine (I) or anti-gp63 antibody (J) were used to score their effect on exosome-dependent inhibition of miRNA-mediated repression. Normal IgG was used as a control. Data represent mean ± SEM. Significance level, ^∗^p < 0.05, ^∗∗^p < 0.001, ^∗∗∗^p < 0.0001). Ld, *Leishmania donovani*. See also Figure S3 and Movie S1.

The in vivo experiments described above confirmed the lowering of miR-122 in *L. donovani*-infected mouse liver. In liver, *L. donovani* infects the tissue macrophage Küpffer cells, but how the infection of Küpffer cells induces miR-122 downregulation in hepatocytes is not known. To understand the mechanism of hepatic miR-122 downregulation in infected animals, we used human Huh7 hepatoma cells that express miR-122. Huh7 cells were transfected with a *Renilla* luciferase reporter either with no miRNA binding sites (RL-con) or with three imperfect miR-122 binding sites (RL-3xbulge-miR-122; Figure 4A) to assess the miR-122 activity in Huh7 cells before and after interaction with *L. donovani*.

To imitate the liver microenvironment, we cocultured Huh7 cells (expressing RL reporters) with isolated human peripheral blood mononuclear cells (PBMC) in the presence and absence of *Leishmania* parasites at different cell-to-parasite ratios; the monocyte in PBMC served as the parasite host and Huh7 cells as hepatocytes. There was essentially no change in miR-122 activity when Huh7 and PBMC were cultured in the absence of the parasite (data not shown). For a fixed number of Huh7 cells, with an increase in PBMC-to-parasite ratio, there was a moderate change in miR-122-mediated repression in Huh7 cells (Figure 4C). It is possible that a host secretory factor, probably a pro- or anti-inflammatory cytokine, secreted by the infected macrophage/monocyte cells changes the hepatic microenvironment that leads to compromised miR-122 activity. To test this hypothesis, we cultured Huh7 cells with *Leishmania*-infected whole PBMC supernatant. No significant changes were observed, even at the highest PBMC-to-parasite ratio (Figure 4B).

From the above experiments it was evident that the parasite itself is the probable candidate to cause the effect on hepatic miR-122. In order to ascertain the importance of direct interaction between Huh7 and *Leishmania* for impairment of miR-122 activity in hepatocytes, we used isolated amastigotes or transformed promastigotes of *L. donovani* to test their effect, if any, on miR-122 activity in human hepatoma cells. Huh7 cells were incubated with *L. donovani* promastigotes at different cell-to-parasite ratios for 24 hr. There was a gradual reduction in repression level with an increasing parasite number per Huh7 cell (Figure 4D). A similar result was obtained when the amastigote form of the parasite was used (Figure 4E). Although upon interaction with the parasite no appreciable change in cellular transcription machineries was observed, a drastic reduction in protein translation was evident in *L. donovani*-treated Huh7 with an overall decrease in proliferation of *L. donovani*-interacting Huh7 cells (Figures S3A–S3C).

Is a parasite-derived secretory factor required for downregulation of miR-122 activity in target cells? Huh7 cells were treated with cell-free *L. donovani* culture supernatant grown at 22°C, the temperature of leishmanial growth at the gut of the sandfly vector. The supernatant could not show any effect on miR-122 activity in Huh7 cells (Figure 4F), but supernatant of the *Leishmania* culture grown at 37°C, the temperature the parasite encounters in mammalian hosts, can reduce miR-122 activity in Huh7 cells (Figure 4F). Thus, *Leishmania*-free culture media at 37°C should contain a factor(s) that can mediate the anti-miR-122 activity in hepatocytes.

### *Leishmania* Glycoprotein gp63 Is Responsible for Reversal of miR-122-Mediated Repression in Huh7

*Leishmania* exosomes, the secretory vesicles released by the parasite, have been documented previously as the carrier of the virulence factors for cellular communication. It is also known that high temperature and low pH increase the rate of exosome secretion by *Leishmania* (Silverman et al., 2010). Hence, we speculate that the exosomes released by the parasites grown at 37°C may contain the key component(s) required for the lowering of miR-122 activity in Huh7 cells. In order to investigate this possibility, we purified exosomes from cell-free *Leishmania* culture supernatant grown at 37°C.

Isolated *L. donovani* exosomes showed reduced miR-122-mediated repression, whereas control exosomes isolated from the culture media kept at 37°C without the parasite showed no difference in miR-122-mediated fold repression compared to untreated cells (Figures 4G and 4H). Previous exploration of the *Leishmania* exosomal cargo proteins revealed that one of the virulence factors, gp63 (a Zn^2+^ dependent metalloprotease), is an abundant protein in *Leishmania* exosomes (Silverman et al., 2010). Preincubation of *Leishmania* exosomes with *o*-Phenanthroline, a Zn^2+^ chelator and an inhibitor of Zn^2+^ metalloproteases, had an inhibitory effect on the ability of *Leishmania* exosomes to reverse miRNA action in Huh7 cells (Figure 4I). A rabbit polyclonal antibody raised against *L. donovani* gp63 was used to block its activity in vitro (Bhowmick et al., 2008). Exosomes preblocked with this anti-gp63 antibody could not prevent the miRNA repression, whereas pretreatment with normal rabbit immunoglobulin G (IgG) failed to reverse the inhibitory role of leishmanial exosomes on miR-122 activity in Huh7 cells (Figure 4J). Overall, these experiments suggest that gp63 glycoprotein, a *Leishmania* exosomal component, is responsible for the anti-miR122 activity observed in Huh7 cells.

### Internalization of *Leishmania* Exosomes Is Essential for miR-122 Downregulation in Hepatocytes

Entry of *L. donovani* or parasite-derived exosomes into host cells is well documented (Silverman et al., 2010). No such direct evidence is reported for cellular entry of either intact *Leishmania* or *L. donovani* exosomes in liver parenchymal cells. From the previous experiments it was evident that the exosomes caused downregulation of miR-122 activity in human hepatoma cells, but it was not clear whether the internalization of the exosomes in heptocytes is required or if its interaction with the membrane of Huh7 cells is sufficient for lowering miR-122 activity. Immunofluorescence analysis showed internalization of gp63 in Endo-GFP-expressing Huh7 cells that were treated with *L. donovani* exosomes. In exosome-treated Huh7 cells, the Endo-GFP-tagged early endosomes were either colocalized or showed proximal localization with gp63 (Figure S3D). Three-dimensional reconstitution of the z-stacked images obtained with a spinning disc confocal microscope was used to confirm the colocalization of the gp63 signal with early endosomes (Figure S3E; Movie S1). Therefore, the entry pathway of *Leishmania* gp63 to Huh7 cells is possibly endosomal. In subsequent experiments with Endo-GFP-expressing Huh7 cells treated with siRNA against dynamin 2, a GTPase involved in membrane trafficking of vesicles (Durieux et al., 2010) showed no internalization or co- or proximal localization of gp63 with Endo-GFP inside the hepatocytes. GFP-tagged endosomes in cells treated with control siRNA showed dynamin 2 colocalization with Endo-GFP and gp63 (Figures S3F and S3G). Downregulation of gp63 internalization in dynamin 2-compromised cells also reduced the effect of exosomes on miR-122-mediated repression in Huh7 (Figure S3H).

Do intact parasites internalize inside the hepatocytes? When incubated with live *L. donovani*, parasitic nuclear DNA stained with DAPI and proximally localized with gp63 signals in intact parasites was absent in Huh7 cells but visible in murine peritoneal macrophages. This suggests an absence of intracellular parasites in infected hepatic cells (Figure S4A and S4B).

In infected mouse liver, immunofluorescence staining for gp63 identified infected regions with elevated gp63 signal that faded out with distance from the zone of infection. Interestingly, apart from the intracellular signals of gp63 inside the hepatocytes surrounding the infected region, strong signals from gp63-positive vesicles/bodies along the intercellular space between hepatocytes were evident (Figure S4C).

### Accumulation of Pre-miR-122 and Failed miRNP-122 Formation Accounts for the Reduced miR-122 Activity in *L. donovani*-Treated Huh7 Cells

**Figure 5:**
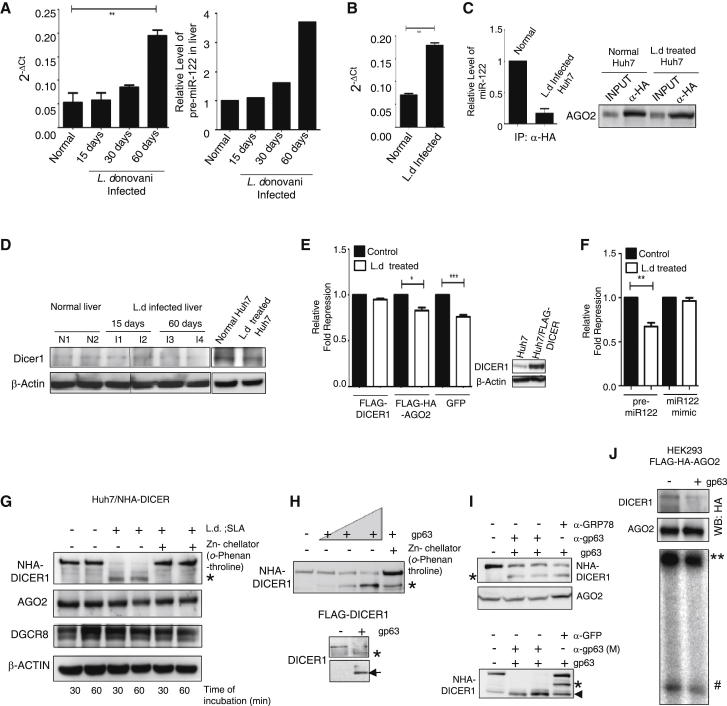
*L. donovani* Downregulates DICER1 and Prevents miRNP Formation in Huh7 Cells (A) *L. donovani* increases pre-miR-122 levels in livers of *L donovani*-infected animals. RNA isolated from the livers of BALB/c mice after 15, 30, or 60 days infected were subjected to real-time analysis, and β-actin mRNA levels were used for normalization. n = 6 for each group. (B) pre-miR-122 levels in Huh7 cells before and after treatment with *L. donovani* promastigotes for 24 hr. Real-time quantification for pre-miR-122 was performed. SDs were from three independent measurements. (C) Association of miR-122 with AGO2 in Huh7 cells interacted with *L. donovani*. RNA was isolated from immunoprecipitated (IP) materials from normal and infected cell lysates, and miR-122 levels were quantified by real-time quantification and normalized against immunoprecipitated AGO2. Quantification data are the mean values obtained from three IP reactions. Western blot was used to detect AGO2 in immunoprecipitated materials. (D) Dicer1 levels in *Leishmania*-infected animal livers. Protein extracts were prepared from normal and infected mouse livers (15 or 60 days p.i.) and western blotted for Dicer1. Huh7 cells treated with *L. donovani* also showed reduction in DICER1 levels. (E) Exogenous expression of DICER1 inhibits *L. donovani*-mediated inhibition of miR-122 activity in Huh7 cells. Cells expressing RL reporters were transfected with plasmids encoding FLAG-DICER1, FLAG-HA-AGO2, or GFP, and the effects of *L. donovani* on miR-122 activity was scored. DICER1 overexpression was confirmed by western blot. (F) Unlike pmiR-122 overexpressed cells, cells transfected with miR-122 mimics can escape *L. donovani*-mediated repression of miR-122 activity. miR-122 repressive activity was measured in normal and *L.donovani*-interacting Huh7 cells expressing pre-miR-122 or miR-122 mimic. (G) DICER1 can be specifically cleaved by the Zn-metalloprotease present in the SLAs. Huh7 extracts were incubated with SLA in the presence and absence of Zn-chelator for 30 or 60 min at 37°C and were western blotted for HA-DICER1, AGO2, and DGCR8. β-actin was used as loading control. (H) *L. donovani* surface protease gp63 cleaves DICER1 and generates two fragments in vitro. Lysates of HEK293T cells expressing NHA-DICER1 were incubated with an increasing concentration of purified gp63 in the absence and presence of Zn-chelator, and cleaved product was visualized by western blot using an HA-specific antibody. It detected a shortened DICER1 band that was ∼180 KDa after cleavage of full-length protein by gp63. Digestion of FLAG-DICER1 also generated a 180 KDa fragment (marked by ^∗^). The N-terminal and short C-terminal half (marked by an arrowhead) of DICER1 were both detected with a DICER1-specific antibody. (I) Treatment with specific polyclonal and/or monoclonal antibody modify gp63 activity in vitro. Blocking of gp63 with a polyclonal antibody raised against the recombinant protein reduces DICER1 cleavage activity. Anti-GRP78 antibody was used as control (upper panel). Pretreatment of gp63 against a monoclonal antibody augments its activity in DICER1 cleavage assay (lower panel). Anti-GFP antibody was used as control. The arrowhead denotes a secondary cleaved product increased in the presence of anti-gp63 monoclonal antibody. (J) The cleavage of AGO2-associated NHA-DICER1 by gp63 and its effect on pre-miRNA processing. Extracts of HEK293 stably expressing FLAG-HA-AGO2 and transfected with NHA-DICER1 were digested with purified gp63 and subsequently FLAG-HA-AGO2 was immunoprecipitated. FLAG-HA-AGO2-associated DICER1 was detected by western blot, and pre-miR-122 processing activities associated with immunoprecipitated materials were quantified. ^∗∗^ denotes the pre-miR-122 substrate; # denotes the mature miR-122 formed; WB, western blot. Data represent mean ± SEM. See also Figure S4.

Interestingly, unlike the mature form, the pre-miR-122 increased in both parasite-infected mouse livers and *L. donovani*-treated Huh7. Real-time quantification of pre-miR-122 confirmed accumulation of the precursor form with progressive infection, which augmented to 3-fold higher than its normal level at 60 days p.i. (Figure 5A). The pre-miR-122 also increased 2.5-fold in Huh7 cells after its interaction with *L. donovani* (Figure 5B).

Why should the precursor accumulate in *L. donovani*-interacting hepatic cells? In eukaryotes, pre-miRNAs are cleaved and processed by an RNase III enzyme, DICER1. This protein processes the pre-miRNAs and loads the processed mature miRNA to AGO proteins to form active miRNPs (Bartel, 2004). When the AGO2 protein was immunoprecipitated from normal and infected Huh7 cell lysates, we documented a 6-fold reduction of miR-122 association with immunoprecipitated AGO2 in *L. donovani*-treated Huh7 (Figure 5C). The observation was similar when miR-16 association with AGO2 was measured (data not shown). These results signify an impaired processing of precursor miRNAs as a mechanism that leads to reduced miRNP formation, which accounts for lowered miRNP activity in *L. donovani*-interacting hepatic cells.

### gp63 Cleaves DICER1 to Prevent Effective miRNP Formation and Inhibits miR-122 Activity in Human Hepatocytes

From the previous experiments it was evident that AGO2 failed to get loaded with miR-122 and form active miRNP in *L. donovani*-exposed hepatic cells. Hence, it was essential to look at the status of Dicer1 in infected tissue samples. We detected a low level of Dicer1 in infected livers after 60 days p.i. as well as in Huh7 treated with *L. donovani* (Figure 5D). Overexpressed DICER1 in Huh7 cells inhibited the derepression of miR-122 activity by *L. donovani* (Figure 5E). Interestingly, when a miR-122 mimic that does not require the DICER1 processing step was transfected in Huh7 cells, the inhibition of miRNA activity by *Leishmania* was lost. However, in Huh7 cells expressing exogenous pre-miR-122 that required DICER1 processing to generate miRNP-122, *L. donovani*-mediated reversal of miR-122 activity was unaffected (Figure 5F). These results further confirm Dicer1 as the primary target of *Leishmania* to downregulate miR-122 activity in mammalian liver. We anticipate degradation of DICER1 by a *Leishmania*-derived factor in target hepatic cells. We observed that HA-tagged DICER1 expressed in Huh7 cells was cleaved in the presence of *Leishmania* lysate when incubated in vitro (Figure 5G). The cleavage of DICER1 was primarily by a Zn-metalloprotease, as the DICER1 degradation was prevented in the presence of *o*-Phenanthroline (Figure 5G). This experiment indicates that gp63, the most abundant Zn-metalloprotease present in leishmanial exosomes, is responsible for DICER1 cleavage. That was further tested when purified gp63 was incubated with DICER1 in an in vitro reaction. Purified gp63 cleaved DICER1 and generated the same fragments obtained with the leishmanial extract. A cleaved N-terminal fragment of 180 kDa was generated along with a shorter C-terminal half (Figure 5H). The specificity of gp63 in Dicer1 cleavage reaction was further confirmed in experiments in which blocking with a polyclonal antibody reduced the activity of purified gp63. Interestingly, the monoclonal antibody treatment augmented the activity of gp63 (Figure 5I), possibly due to a change in its conformation upon antibody binding as reported earlier for few other enzymes (Benito et al., 1996; Cooper and King, 1986). DICER1 associates and transfers the processed mature miRNA to AGO2 to form miRNPs. In cell extract treated with gp63, we also found reduced association of AGO2 with full-length DICER1, resulting in decreased pre-miR-122 processing (Figure 5J). Therefore, *L. donovani* targets DICER1 to prevent active miRNP formation.

### Restoration of Dicer1 Expression in Parasite-Infected Livers Rescues miR-122 Expression and Reduces Liver Parasite Burden

**Figure 6:**
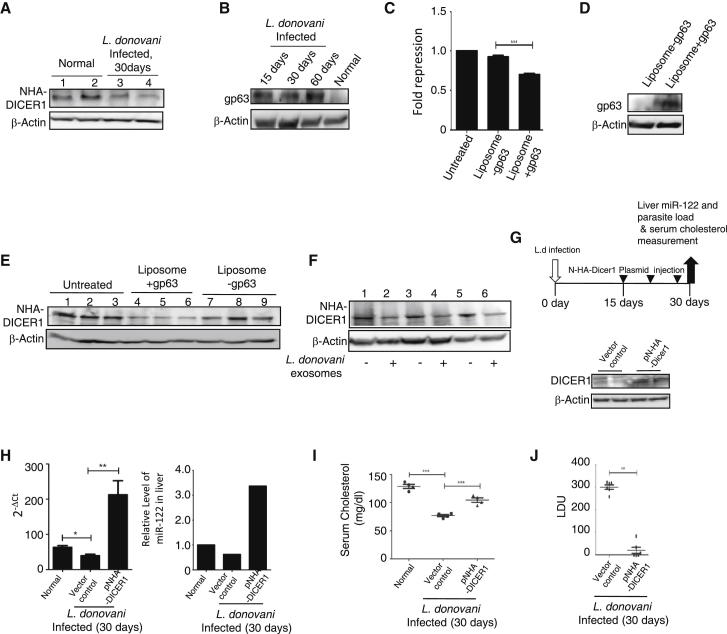
Restoration of Dicer1 Expression Rescues Serum Cholesterol Levels and Lowers Hepatic Parasite Load in *L.donovani*-Infected Mouse Livers (A) Dicer1 expression in *L. donovani*-infected mouse liver. Expression of NHA-DICER1 in the livers of control or infected (30 days p.i.) *L. donovani* BALB/c mice three days postinjection of NHA-DICER1 expression plasmid through tail vein. (B) Increase of gp63 in infected mouse liver extracts western blotted for gp63. (C) Effects of liposomal delivery of gp63 on miR-122 activity in Huh7 cells. Liposomal formulations with or without purified gp63 were delivered to cells expressing miR-122 reporter, and fold repression was calculated. (D) Treatment with gp63-containing liposomes delivers the protein to animal liver. Western blot detection of gp63 was done in liver lysates from control and liposome-treated animals using an anti-gp63 antisera. (E) Effect of gp63-containing liposomes on liver DICER1. Adult BALB/c mice were injected first with NHA-DICER1-expressing plasmids and then with liposomes (100 μl liposome suspension with or without 100 μg of purified gp63). Liver lysates were prepared after 24 hr of liposome injection and western blotted for NHA-DICER1. (F) Effect of *L. donovani* exosome treatment on the mouse liver DICER1 level. Adult BALB/c mice expressing NHA-DICER1 protein were injected through the intracardiac path with *L. donovani* exosomes secreted by 5 × 10^7^ parasites. After 24 hr of exosome treatment, liver lysates were prepared and western blotted for the NHA-DICER1. (G) Expression of NHA-DICER1 increases cellular Dicer levels in mouse livers. Schematic representation of protocol of NHA-DICER1 expression in BALB/c mice livers (upper panel). Endogeneous Dicer1 levels in both normal and NHA-DICER1 expression-plasmid-injected mouse livers after 3 days of plasmid injection (Lower panel). (H–J) NHA-DICER1 can prevent infection progression in mice. *L. donovani*-infected animals were injected with NHA-DICER1 expression plasmids and sacrificed as per the schedule in (G). Relative miR-122 expression (H), serum cholesterol level (I), and hepatic parasite load (J) were compared between NHA-DICER1 plasmids injected and control groups. For all experiments, n was either 4 or 6, and representative western blots were shown. For all western blot experiments β-actin was used as loading control. Data represent mean ± SEM. ^∗∗∗^p < 0.0001, ^∗∗^p < 0.001, ^∗^p < 0.01. See also Figure S5.

When expressed exogenously, NHA-DICER1 expression was found to be low in *L. donovani*-infected mouse livers (Figure 6A). This observation was consistent with the prediction of Dicer1 as a target that *L.donovani* degrades to downregulate miR-122 in infected mouse liver. With infection, the level of gp63 was increased in mouse livers (Figure 6B). To confirm that gp63 can downregulate Dicer1 in vivo, purified gp63 from *L. donovani* extract was entrapped in liposomes before being delivered to Huh7 cells. Interestingly, a substantial decrease in miR-122 activity was noted in cells receiving gp63-containing liposomes (Figure 6C). These liposomes were found to be more enriched in mouse livers 24 hr after in vivo delivery (Figure S5A) and were subsequently used for in vivo administration of gp63 in BALB/c mice expressing NHA-DICER1. Delivery of gp63 to liver was confirmed by western blot (Figure 6D). Interestingly, the level of NHA-DICER1 was significantly low in the livers of animals treated with gp63-containing liposomes (Figure 6E). During infection, exosomes may be the vehicles for gp63 transfer to hepatocytes (Figures S3 and S4C). Delivery of purified *L. donovani* exosomes downregulated DICER1 in animal livers (Figure 6F).

To see whether DICER1 overexpression can clear *L. donovani* from infected mouse liver, we administered NHA-DICER1-expressing plasmids (Figure 6G). Excess Dicer1 in liver increased liver miR-122 expression and restored the serum cholesterol level (Figures 6H and 6I). This was accompanied with a drastic reduction in liver parasite load (Figure 6J). Importantly, no apparent change in liver cell morphology, tissue integrity, or production of serum albumin was documented (Figures S5B and S5C).

## Discussion

In *L. donovani*-infected mouse liver, almost 20% of the total genes showed an altered expression. Several genes of cholesterol metabolism that are indirect targets and reciprocally regulated by miR-122 (Elmén et al., 2008) were downregulated along with miR-122 in *L. donovani*-infected liver. Animals manipulated to have excess miR-122 levels in their livers showed resistance to *L. donovani* infection (Figures S2B–S2E). This observation further supports the existence of a balance of liver miR-122 and serum cholesterol that get influenced and exploited by *Leishmania* parasites in infected mammals. Therapy against VL is still a big challenge, and recently, the emergence of drug resistance has added to the problem (Croft et al., 2006). Overexpressing miR-122 in infected liver tissue showed appreciable clearance of hepatic parasite burden with a recovery of serum cholesterol. Therefore, the therapeutic potential of miR-122 alone and in combination with cholesterol can open up avenues to combat this deadly disease.

*L. donovani* infection in mice shows a decrease in liver DICER1 expression accompanied by a concomitant increase of its substrate pre-miR-122. Pre-let-7a RNA also showed similar accumulation in infected mouse livers (data not shown). Therefore, this might be a general mechanism for *Leishmania* to lower the activities of miRNAs in the host cell. Interestingly, *L. donovani* infection also leads to the lowering of Dicer1 and let-7a miRNA in infected mouse macrophages (J.G., Y. Chakraborty, and S.N.B., unpublished data).

Inactivation of specific miRNA by virus-encoded target RNAs or virus-encoded miRNA binding proteins in virus infected cells has been reported previously (Belair et al., 2011; Cazalla et al., 2010; Libri et al., 2012). Here, we report a leishmanial protease gp63 that targets Dicer1 to reduce miRNA activity in hepatic cells. But how the gp63, produced by the Küpffer cell resident parasites, gets transferred to the neighboring target hepatocytes is an unsolved issue. The *Leishmania* exosomes may act as the vehicles for transport of gp63 cargo to the hepatocytes in mouse liver. This idea was supported by downregulation of DICER1 expression in liver upon exposure to *Leishmania* exosomes (Figure 6F). Interestingly, mammalian macrophages are also known to secrete exosomes and microvesicles that are used for intracellular transport and signal communication (Bhatnagar et al., 2007). Therefore, it may be possible that parasite-released gp63 get trapped within the exosomes secreted by the host cells and get delivered to hepatocytes to target Dicer1 and reduce miRNA activity. Immunofluorescence detection of gp63 in infected livers was suggestive of propagation of this protease through the intercellular spaces in *L.donovani*-infected livers.

Overall, the present study deals with an interesting connection between altered lipid metabolism during *L. donovani* infection and liver miR-122 levels. We report an alteration of a miRNA in a parasite-infected tissue that can account for an important metabolic change necessary for pathogenesis. This also documents a unique strategy that the parasite evolved to combat regulatory RNA function in host cells. With human hepatic cells, we have shown that leishmanial metalloprotease gp63 targets DICER1 in human hepatic cells to reduce miR-122 activity. A similar mechanism is operative for downregulation of miRNA activity in *L. donovani*-infected mouse liver. It will be interesting to investigate whether other members of the Trypanosomatidae family have evolved a similar mechanism for their survival in infected host.

## Experimental Procedures

### Infection Delivery and Postinfection Analysis

*L. donovani* strain AG83 (MAOM/IN/1083/AG83) was used to infect 4- to 6-week old BALB/c mice with 2^nd^ passage promastigotes or amastigotes (10^7^ parasites/animal) by the intracardiac route. After stipulated days of infection, blood serum was collected and liver extract was used for RNA or protein analysis. Liver sections were used for histological examination, and parasite count was accomplished by microscopic evaluation of Giemsa-stained tissue imprints. Leishman-Donovan unit (LDU) = number of amastigotes/1,000 nucleated cells × weight of spleen or liver (g).

All animal experiments approved by the institutional animal ethics committee were carried out following the national guidelines set by the government of India.

### Parasite Transformation, Amastigote Isolation, Culture Supernatant Collection, and Soluble Leishmanial Antigen Preparation

Amastigotes were isolated from *L. donovani*-infected golden hamsters. They were either used directly or transformed to promastigotes and maintained until the second to fourth passage cultures before the promastigotes were used. Culture supernatants used for in vitro assays were collected from cultures grown at 22°C and 37°C. Soluble leishmanial antigen (SLA) was prepared after sonication of the promastigotes as described elsewhere (Saha et al., 1991).

### Whole-Genome Microarray Analysis

Microarray analysis was done on an Illumina Sentrix Chip (Mouse WG-6 v2.0 Beadchip) using total RNA isolated from normal mouse livers and *L.donovani*-infected mouse liver after 60 days. TCGA Catalyzing Genomics (New Delhi, India) performed the analysis, and the data were analyzed after background noise correction and normalization. Differential expression analysis was done using Illumina custom algorithm. All the data have been filtered based on detection; differential p value ≤ 0.05. The whole microarray data have been uploaded in Gene Expression Omnibus database (accession number GSE38985).

### Exogenous Expression of miR-122 and Dicer in Mouse Liver

Exogenous expression studies were conducted according to a published hydrodynamic injection strategy (Tada et al., 2006; Andrianaivo et al., 2004). To overexpress miR-122 or NHA-DICER1 in mouse livers, pmiR-122, the pre-miR-122 expression plasmid, or the pCIneoNHA-DICER1 plasmid was injected through the tail vein of mice at a dose of 25 μg plasmid DNA dissolved in 100 μl saline. Animals weighing 20–25 g were used and sacrificed at 1, 3, 5, and 7 days postinjection to determine liver miR-122 levels. For NHA-DICER1 expression examination, animals were sacrificed after 3 days. In experimental animals, injections were delivered to *L. donovani*-infected mice in three individual doses starting from 15 days p.i. at intervals of 6 days. Mock plasmid-treated (100 μl each) animals were kept as sham-treated controls. Animals were sacrificed at 30 days p.i. along with the control group of animals, and serum lipid profile, liver parasite burden, and miR-122 expression levels were determined.

### Cell Culture, Transfection, Treatment, and Luciferase Assays

Huh7 cells were cultured, and all transfections were performed using Lipofectamine 2000 (Invitrogen) following manufacturer’s instructions. *Renilla* luciferase (RL) and firefly luciferase (FL) activities were measured using a Dual-Luciferase Assay Kit (Promega) measured on a VICTOR X3 Plate Reader system (PerkinElmer), and fold repression was calculated.

### Northern and Western Blot Analyses

Total RNA isolated from mouse liver was electrophoresed in 15% denaturing TBE-Urea polyacrylamide gel and transferred to Immobilon-NY+ transfer membrane. It was probed with 5′ end ^32^P-labeled oligonucleotide probes against hsa-miR-122 at 37°C for 16 hr in a hybridization oven. Phosphoimaging was performed with a Cyclone Plus Storage Phosphor System (PerkinElmer). Liver tissue proteins were extracted in RIPA buffer, separated using SDS-PAGE, transferred to polyvinylidene fluoride (PVDF) membrane, and western blotted for different proteins using specific antibodies. β-actin was used as a loading control. gp63 was detected in liposome-treated mouse livers by western blot against antisera to gp63.

### Real-Time Quantitative RT-PCR

The miR-122, miR-16, and U6 levels were quantified with a real-time RT-PCR detection assay kit (Applied Biosystems) from total RNA following the manufacturer’s instructions. mRNA and pre-miRNA quantifications were done using the SYBR Green Real-Time PCR Assay Kit (Invitrogen). The results were normalized against 18S ribosomal RNA (rRNA) for mRNAs and against β-actin for pre-miR-122.

### Exosome Isolation and Treatment

Stationary-phase *L. donovani* promastigote or amastigote (∼5.0 × 10^7^ cells/ml) culture was kept at 37°C overnight. As a control, M199 medium supplemented with 10% fetal calf serum (FCS), but without the parasites, was used. Exosomes were isolated as described earlier (Raposo et al., 1996) with minor modifications. For further treatment, exosomes (100 μg protein per assay) were incubated for 1 hr at room temperature with *o*-Phenanthrolin or 4°C overnight with anti-gp63 antibody before the exosomes (treated or untreated) were used in subsequent assays with Huh7 cells.

### Immunoprecipitation

For immunoprecipitation (IP) reactions, HA-AGO2-transfected cells were lysed and incubated with anti-HA (Roche) bound protein G agarose beads overnight at 4°C. As control, anti-GFP bound protein G agarose beads (Invitrogen) were used. After that, the beads were washed thrice with IP buffer, and the bound proteins were analyzed by western blot. Half of the beads separated during washing steps were used for RNA extraction.

### In Vitro DICER1 Cleavage Assay

For in vitro cleavage assay, NHA-DICER1 expressing Huh7 cell extract was incubated with *L. donovani* SLA or purified gp63 in buffer containing 10 mM Tris-HCl (pH 7.5), 1mM DTT, 100 mM KCl, and 1× protease inhibitor cocktail. Incubation was done at 37°C for 30 or 60 min. The reactions were stopped with SDS sample buffer. For the *o*-Phenanthrolin treatment, SLA was preincubated with 10 μM *o-*Phenanthrolin for 30 min on ice before being used for the assay. For testing the purity of isolated gp63, a mouse monoclonal and a polyclonal anti-gp63 antibody were used to block gp63 activity at 1:10 and 1:25 dilutions overnight. Anti-GFP and anti-GRP78 were used at the same dilutions as the control. The samples were run in 8% SDS-PAGE, and western blot was performed using anti-HA antibody to detect NHA-DICER1.

### In Vitro Pre-miRNA Processing Assay

The NHA-DICER1 expressing HEK293 cell lysate was incubated with purified gp63 followed by immunoprecipitation with anti-FLAG M2 affinity gel. Washed beads were used for pre-miR122 processing assay with 10 nM pre-miR122, and the products were analyzed on a 12% denaturing (8 M Urea) polyacrylamide gel and visualized in a phosphoimager.

### Introduction of Liposomes and *L. donovani* Exosomes in BALB/c Mice

Adult BALB/c mice were injected with NHA-DICER1-expressing plasmids via tail vein. After 2 days, liposomal formulations or leishmanial exosomes were injected through the intracardiac route. After 24 hr, mice were sacrificed, liver lysates were prepared, and exogenous DICER1 levels were detected by western blot analysis using anti-HA antibody. For liposome treatment of Huh7 cells, luciferase reporter transfected cells were treated with 200 μl liposome suspension.

### Other Experimental Procedures and Details

Additional experimental procedures and other essential details on plasmids, oligos, and antibodies used have been provided as Supplemental Experimental Procedures.

## Accession Numbers

The Gene Expression Omnibus (GEO) accession number for the whole-genome microarray expression data reported in this paper is GSE38985.

## Supplemental Information

Document S1. Figures S1–S5, Tables S1–S3, Movie S1, and Supplemental Experimental Procedures

Table S2. Microarray Analysis Shows Substantial Changes in Expression of Lipid Metabolizing Genes, Related to Figure 2 RNA samples from normal animal livers and those 60 days p.i. were subjected to a whole-genome microarray analysis. Lipid metabolic genes showing a fold change ≥ 2 are listed with fold changes in their expression in infected samples against their expression levels in normal livers. Type 1 genes are downregulated in infection, whereas type 2 genes are upregulated.

Movie S1. Proximal Association or Colocalization of Internalized gp63 with GFP-Tagged Endosomes in *L. donovani* Exosome-Treated Huh7 Cells, Related to Figure 4 z-stacked merged images of Endo-GFP-expressing Huh7 cells treated with *L. donovani* exosomes and stained for gp63 were captured with an ANDOR Spinning Disc Confocal microscope and processed with Nikon NIS Element AR 3.2 software to get the 3D view of the cells. The 3D rotational views were captured at a rate of 30 frames/sec to create the movie. Endo-GFP is in green and gp63 is in red. DAPI was used to stain the nucleus.
