# Supplementary material for: Leishmania donovani Targets Dicer1 to Downregulate miR-122, Lower Serum Cholesterol, and Facilitate Murine Liver Infection
Source: Cell Host Microbe. 2013 Mar 13;13(3):277–88. doi: 10.1016/j.chom.2013.02.005 (PMC3605572; doi:10.1016/j.chom.2013.02.005)
Supplement: Document S1. Figures S1–S5, Tables S1–S3, Movie S1, and Supplemental Experimental Procedures [file mmc1.pdf]

Cell Host & Microbe, Volume *13*

Supplemental Information

Leishmania donovani **Targets Dicer1 to Downregulate miR-122, Lower Serum Cholesterol, and Facilitate Murine Liver Infection**

June Ghosh, Mainak Bose, Syamal Roy, and Suvendra N. Bhattacharyya

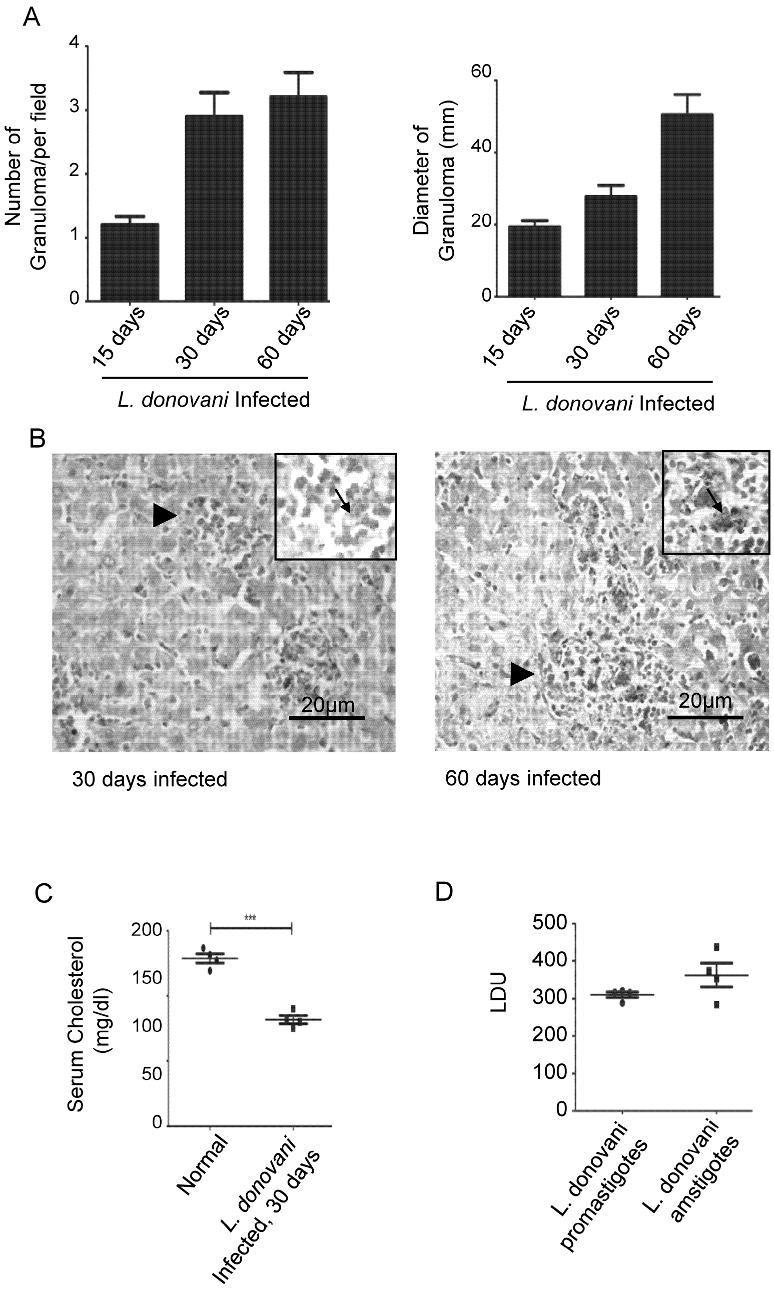

Figure S1. *L. donovani* Infection Affects Granuloma Formation in Infected Mouse Liver while Infection with Amastigotes Also Showed Reduced Serum Cholesterol, Related to Figure 1

(A, B) Change in number and average diameter of granuloma during *L. donovani* infection in mouse liver. Paraffin sectioning was done with paraformaldehyde fixed mouse liver and Hematoxylin-Eosin staining was performed. Number of granuloma visible per field and their average diameter were plotted for 15, 30 and 60 days infected mouse liver sections (A). Data is represented as mean +/- SEM. Representative microphotographs taken at 40X magnifications from 30 and 60 days infected mouse liver sections stained

with Hematoxyline-Eosin. The zoomed part shows the parasite loaded tissue macrophages. Arrowheads indicate the granulomas while arrows are showing parasite loaded tissue macrophages (B).

(C, D) Infection of mouse with *L. donovani* amastigotes reduces serum cholesterol (C) with increase in liver parasite load after 30 days of infection (D)

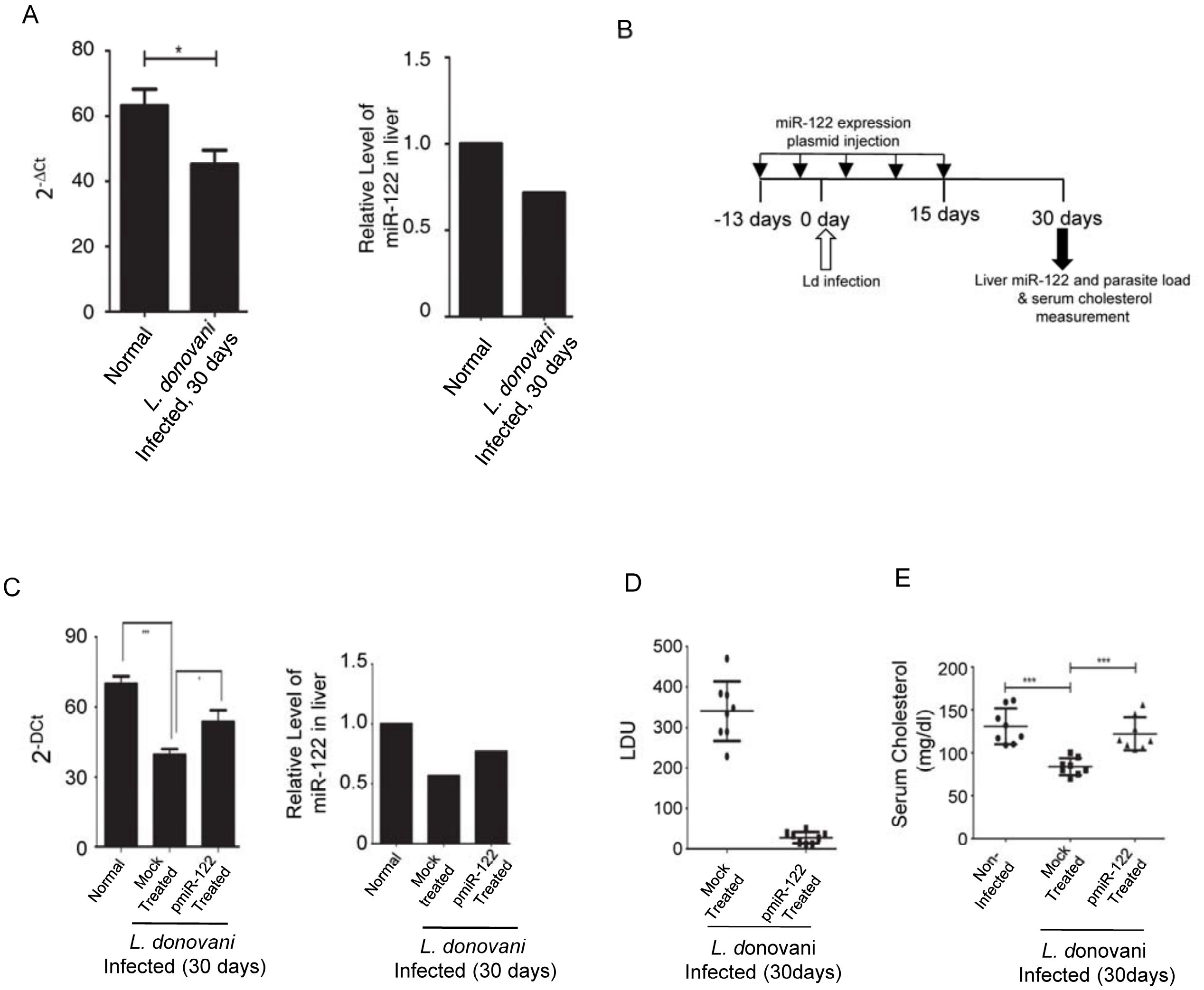

Figure S2. Infection with *L. donovani* Amastigotes Reduces Liver miR-122 while Excess miR-122 Prevents *L. donovani* Infection, Related to Figure 3

(A) Reduction of miR-122 level *in L. donovani* amastigote infected BALB/c mice. Animals were infected with 10^7 *L. donovani* amastigotes and at 30 days post infection mice were sacrificed, blood serum collected and

liver dissected to determine relative changes in liver miR-122 level. Data represents mean ± SEM and n=8. *-p<0.01; ***-p<0.0001.

(B) miR-122 prevents *L. donovani* infection**.** Schematic representation of time course of exogenous miR-122 expression. Infection time is marked by white arrow and the black arrow defines the time of sacrifice 30 days p.i. The Black arrowheads mark the injection time of miR-122 expression plasmids.

(C-E) miR-122 prevents infection in mice. Animals were treated as per the schedule described in panel B and were sacrificed after a month. Relative miR-122 expression (B), serum cholesterol level (D) and hepatic parasite load (E) were compared in pmiR-122 treated and sham treated groups. For all experiments, n=6-8. ***-p<0.0001. L.d; *L. donovani.* Data is represented as mean +/- SEM.

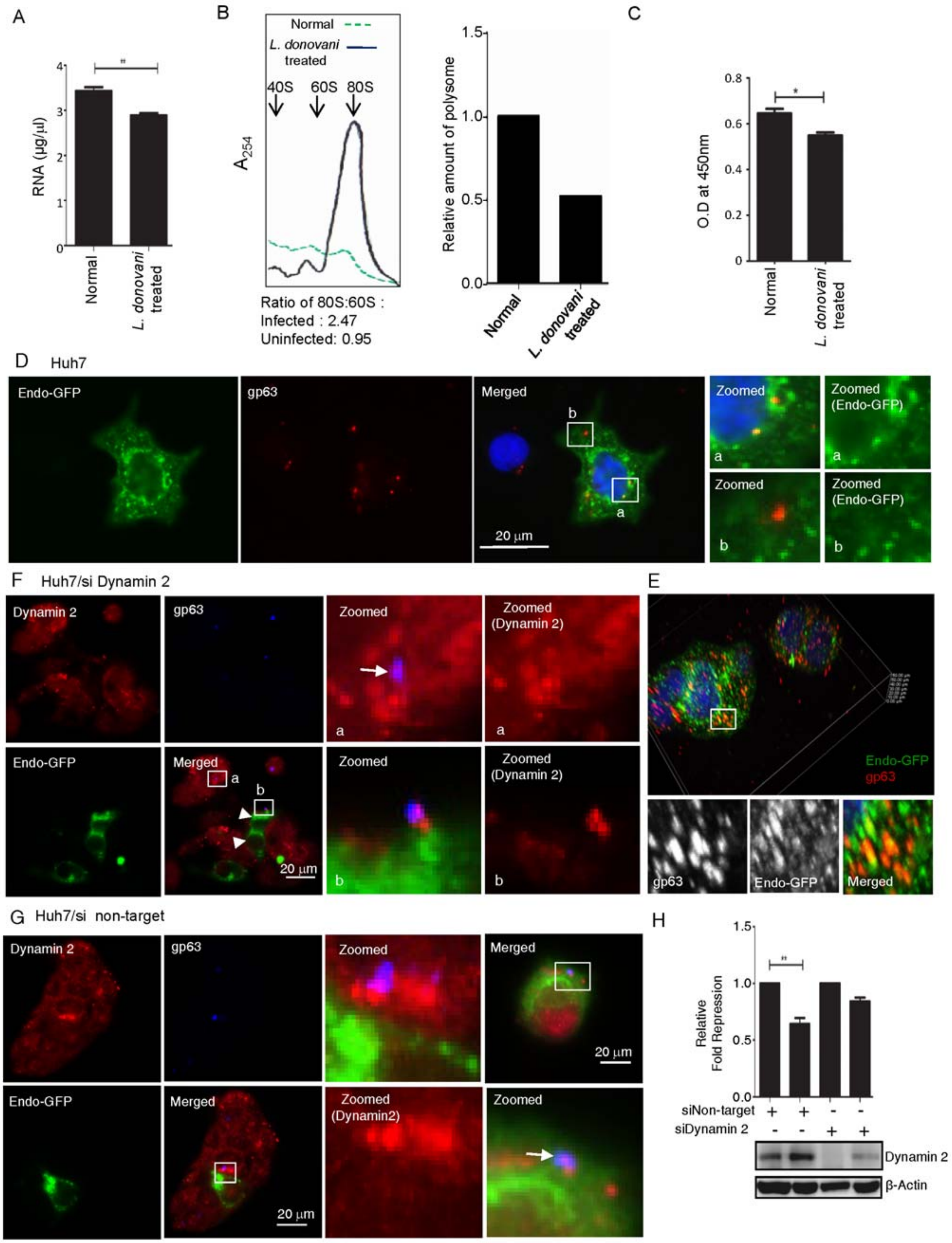

A
RNA (µg/µl)
Normal
L. donovani treated
B
Normal
L. donovani treated
40S 60S 80S
A254
Ratio of 80S:60S :
Infected : 2.47
Uninfected: 0.95
Relative amount of polysome
C
O.D at 450nm
D Huh7
Endo-GFP
gp63
Merged
Zoomed
Zoomed (Endo-GFP)
20 µm
F Huh7/si Dynamin 2
Dynamin 2
Zoomed (Dynamin 2)
E
Endo-GFP
gp63
G Huh7/si non-target
Zoomed (Dynamin2)
H
Relative Fold Repression
siNon-target
siDynamin 2
Dynamin 2
β-Actin

Figure S3. Effect of *L. donovani* Interaction on Huh7 Cells and Dynamin 2-Dependent Internalization of gp63-Containing Exosomes in Huh7 Cells, Related to Figure 4

(A-C) Effects of *L. donovani* infection on Huh7 cells. Cells were treated with the parasite for 24 h and changes in cellular RNA content (A), protein translation status (B) and cell growth rate estimated from BrdU incorporation (C) were determined. In panel B absorbance at 254 nm during gradient fractionation was plotted for first three fraction of normal and parasite treated Huh7 cells. For all experiments, n=3. *-p<0.01; ***-p<0.0001. Data represented as mean +/- SEM.

(D-H) Internalization of gp63 in hepatocytes. Huh7 cells were incubated with *L.donovani* exosomes and internalization of the exosomes was visualized by indirect immunofluorescence using antibodies specific for gp63. Zoomed picture of the marked regions were also shown. Internalization of gp63 in cells expressing Endo-GFP protein (D). To confirm the co- or proximal localization of GFP and gp63-specific signals, a snap view of 3D reconstituted image and its zoomed view are shown in panel E. Endo-GFP tags early endosomes.

(F-G) Defective internalization of *gp63* in cells treated with Dynamin 2 specific siRNAs (siDynamin 2). Huh7 cells were treated with either siDynamin 2 (F) or sinon-target (G) and the internalization of the gp63 was monitored in control and Dynamin 2 depleted cells expressing Endo-GFP. A white arrowhead pointing the Dynamin 2 knockdown cells in panel F and white arrows mark internalized gp63 in control cells. The extreme right column in panel G represents Endo-GFP expressing and control siRNA treated Huh7 cells with internalized gp63 marked by a white arrow.

(H) Effect of depletion of Dynamin 2 on *L. donovani* exosomes mediated downregulation of miR-122 activity in Huh7 cells. Normal or Dynamin 2 depleted Huh7 cells expressing either the RL-con or RL-3xbulge-miR-122, renilla luciferase reporter were grown and treated with *L. donovani* exosomes before the luciferase activities were measured and relative fold repression values calculated. Data represents mean ± SEM. The western blot for Dynamin 2 was done to confirm the depletion of the protein after siRNA treatment. n=3. **-p<0.001.

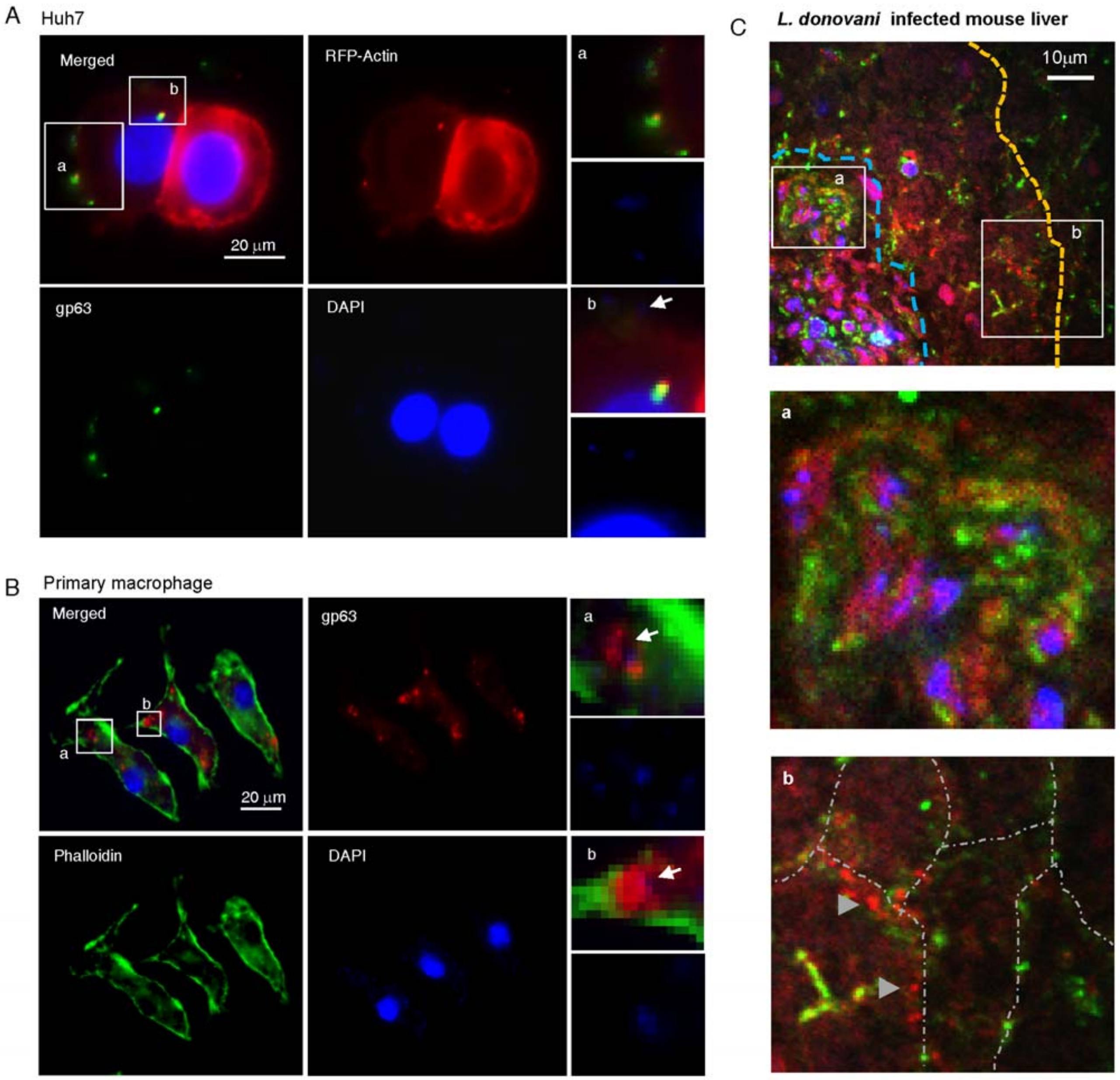

Figure S4. Localization of gp63 in Hepatic and Macrophage Cells Exposed to *L. donovani*, Related to Figure 5

No detection of intact *L. donovani* in parasite treated Huh7. (A) RFP-Actin expressing Huh7 cells were incubated with *L. donovani* and localization of gp63 was visualized by indirect immunofluorescence using antibodies specific for gp63. Zoomed picture of the marked regions are shown in the right column. Huh7 cells express RFP-Actin (red) and gp63 signal is in green (B) Phalloidin coupled to Alexa Fluor®488 was used to stain actin cytoskeleton in primary macrophage where gp63 signal was in red. The Leishmanial genomic DNA and mammalian cell nucleus were stained with DAPI (blue).The white arrows mark the gp63

genomic DNA signals. (C) Localization of gp63 in *L.donovani* infected mouse liver. In immunohistochemical examination of 30 days infected mouse liver section gp63 was detected with anti-gp63 antibody (red) and actin cytoskeleton was stained with Alexa Fluor®488 coupled Phalloidin. DAPI stained the nucleus (blue). The yellow broken line defined the boundary of visible gp63 signal from the zone of infection marked by the blue broken line. The boxed regions were enlarged to show gp63 signal in the zone of infection (a) and in a region far from the infected zone (b) where gp63 localized in intercellular space is marked by white arrowhead and white broken lines mark individual hepatocytes.

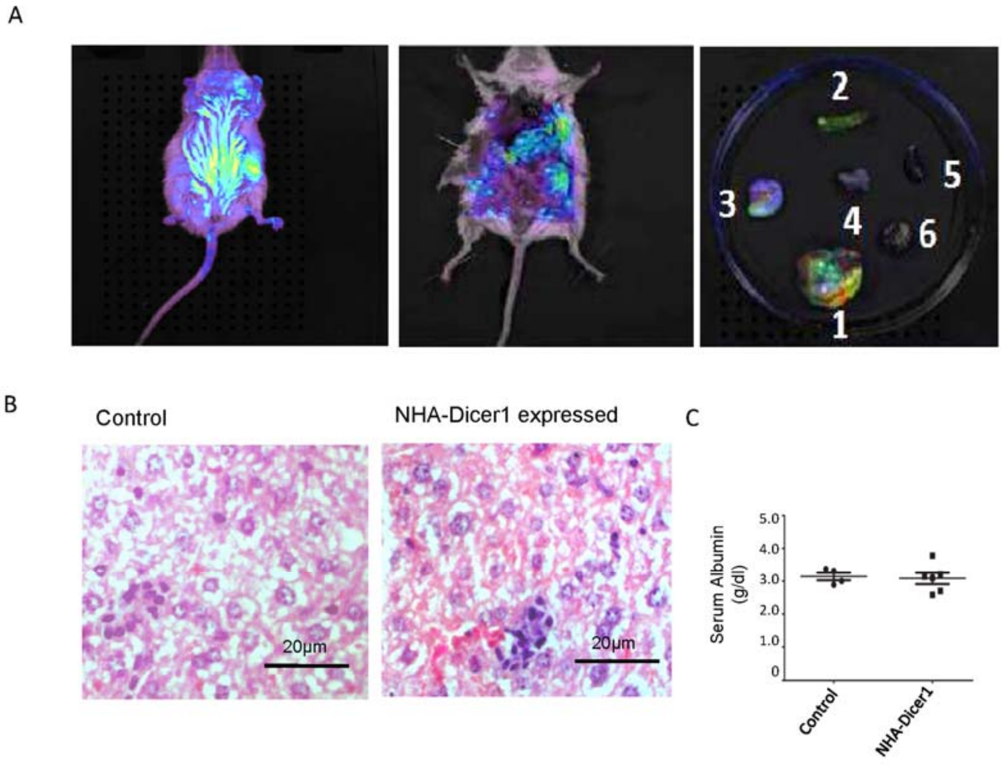

Figure S5. Accumulation of Liposomes in Mouse Liver and the Effect of Dicer Expression on Liver, Related to Figure 6

(A) Live animal imaging of BALB/c mice injected with NBD-cholesterol liposome (100 μl of liposomal suspension via intracardiac route). The left and middle panel represent contour map of NBD-fluorescence in whole animal after 24 h of injection. The right panel show NBD-fluorescence in different organs dissected after animal was sacrificed (1-Liver, 2-Spleen, 3- GI tract, 4- Lymph Node, 5- Heart, 6- Kidney).

(B) Liver histology after NHA-DICER1 expression. Hematoxylin-Eosin staining of paraffin sections of paraformaldehyde fixed liver samples. Representative microphotographs at 40X magnification from control and NHA-Dicer1 expressing mouse liver.

(C) Serum albumin level was measured both for control and NHA-DICER1 expressing mouse liver. n=4 or 6.

Table S1. Liver Parasite Load Shows an Inverse Correlation to Serum Cholesterol in *L. donovani*-Infected Mice, Related to Figure 1

15 days Post Infection

Liver Parasite Load (LDU)	Serum Cholesterol (mg/dl)
180	36
110	96
155	90
205	79
240	86
220	80
160	63
215	77

30 days Post Infection

Liver Parasite Load (LDU)	Serum Cholesterol (mg/dl)
305	87
360	57
235	142
316	34
289	76
317	74
321	76
325	58

60 days Post Infection

Liver Parasite Load (LDU)	Serum Cholesterol (mg/dl)
340	88
400	68
270	83
440	66
365	89
460	83
335	78
380	76

Hepatic parasite load in individual animals were determined at 15, 30 and 60 days post infection by giemsa staining and were tabulated against the corresponding serum cholesterol values.

Table S3. Changes in Expression of Validated miR-122-Controlled Gene in *L. donovani*-Infected Mouse Liver, Related to Figure 3

GENE	Normal(N)	Infected(I)	Fold change(I/N)	Gene name
Mink1	18.8	15.4	**1.220779221**	Misshapen-like kinase 1;Mink1
P4ha1	54.2	54	**1.003703704**	Prolyl 4-hydroxylase subunit alpha-1;P4ha1
Acly	6889.9	8532.2	**0.807517405**	ATP-citrate synthase;Acly
Dgat1	73.3	88	**0.832954545**	Diacylglycerol O-acyltransferase 1;Dgat1
Dgat2	11396.2	14041.3	**0.811620007**	Diacylglycerol O-acyltransferase 2;Dgat2
Fasn	38.6	103.6	**0.372586873**	fatty acis synthase
Ppara	78.8	86.6	**0.909930716**	Peroxisome proliferator-activated receptor alpha;Ppara
Scd1	13737.8	31656.3	**0.43396733**	stearoyl-CoA desaturase (delta-9-desaturase)
Pmvk	370	1002.5	**0.369077307**	Phosphomevalonate kinase;Pmvk
Hmgcr	196.3	570.2	**0.34426517**	3-hydroxy-3-methylglutaryl-coenzyme A reductase;Hmgcr
Rell1	67	104.2	**0.642994242**	RELT-like protein 1;Rell1
Hist1h1c	6811.9	3333	**2.043774377**	Histone H1.2;Hist1h1c
Dscr1	341.9	330.8	**1.033555018**	Calcipressin-1;Rcan1
Mir16	3236.3	3758	**0.861176158**	Glycerophosphodiester phosphodiesterase 1;Gde1
Aldoa	1300.8	1296.2	**1.003548835**	Fructose-bisphosphate aldolase A;Aldoa
Elovl6	310.6	809.5	**0.383693638**	Elongation of very long chain fatty acids protein 6;Elovl6
Fdps	4244.8	5478.5	**0.774810623**	Geranyltranstransferase;Fdps
Idi1	450.3	3742.3	**0.120327072**	Isopentenyl-diphosphate Delta-isomerase 1;Idi1
Sc4mol	4621.8	5072.9	**0.911076505**	C-4 methylsterol oxidase;Sc4mol
Pcsk9	1892.8	2720.3	**0.69580561**	Proprotein convertase subtilisin/kexin type 9;Pcsk9
Cyp51	1045.4	2523.3	**0.414298736**	cytochrome P450, family 51, subfamily A, polypeptide 1
Dhcr7	664.7	1198.1	**0.554795092**	7-dehydrocholesterol reductase;Dhcr7
Acaa1a	17337.6	16353.7	**1.060163755**	3-ketoacyl-CoA thiolase A, peroxisomal;Acaa1a
Acaa1b	21947.5	31610.1	**0.694319221**	
Hsd11b1	20773.8	21032.9	**0.987681204**	Corticosteroid 11-beta-dehydrogenase isozyme 1;Hsd11b1
Acads	1434.1	1331.4	**1.077136848**	Short/branched chain specific acyl-CoA dehydrogenase, mitochondrial;Acadsb
Pla1a	939.8	337.8	**2.782119597**	Acyl-protein thioesterase 1;Lypla1

Microarray analysis revealed a cluster of genes known to be indirect targets of miR-122 which showed a differential expression pattern in *L. donovani* infected than normal mouse liver. Genes are listed with fold change values of in their expression in infected mouse liver.

Supplemental Experimental Procedures

Animal Care

BALB/c mice (4 to 6 weeks old) were obtained from the animal facility of the CSIR-Indian Institute of Chemical Biology. They were housed under conventional conditions with food and water provided ad libitum. All animal experiments were done with necessary prior approval of the institutional animal ethics committee.

Ethics Statement

Use of mice was approved by the Institutional Animal Ethics Committee of CSIR-Indian Institute of Chemical Biology, India. All the experimentations were performed according to the National Regulatory Guidelines issued by the Committee for the Purpose of Supervision of Experiments on Animals, Ministry of Environment and Forest, Govt. of India. All the experiments involving animals were carried out with prior approval of the institutional animal ethics committee.

Reagents, Chemicals, and Buffers

RPMI-1640, DMEM, M-199 medium, fetal calf serum (FCS), goat serum and penicillin-streptomycin mix were purchased from GIBCO, USA. Giemsa Stain, Con A-Sepharose resin, α-methyl-d-mannoside and *o*-Phenanthrolin were from Sigma Aldrich. Bovine serum albumin, Urea, Triton X-100, Sodium deoxycholate, paraformaldehyde, n-octyl-β-D-glucopyranoside, Imidazole, Tris base were obtained from USB Biochemicals. γ-^{32}P-ATP (6.7 Ci/mmol) was obtained from PerkinElmer, Life and Analytical Sciences, USA when Tween 20, DTT, formamide were from Calbiochem and Trizol reagent was from Invitrogen. let-7a mimic and miR-122 mimic were from Ambion Life technologies. sinon-target was from Invitrogen and

siDynamin 2 was from Sigma Aldrich. Phosphaditylcholine was obtained from Avanti Polar Lipids and Cholesterol and NBD-Cholesterol were purchased from Sigma Aldrich. IP buffer contain 20 mM Tris-HCl, pH 7.5, 150 mM KCl, 5 mM $MgCl_2$, 1 mM DTT.

Cell Lines

Huh7, a human hepatoma cell line and HEK293, human embryonic kidney cell line with SV40 T antigen were cultured in Dulbecco's modified Eagle's medium (DMEM) supplemented with 10% heat-inactivated FBS (fetal bovine serum), 100 units/ml penicillin and 100 µg/ml streptomycin at 37°C under humidified air containing 5% CO_2.

Primary Cell Isolation

Human Peripheral Blood Mononuclear Cells (hPBMC) were obtained by gradient centrifugation of heparinized venous blood over Ficoll-Paque by centrifugation at 800xg at room temperature for 30 min in a swing bucket rotor. The intermediate white ring of cells were collected into a fresh tube, washed with PBS and plated according to the requirement. Mouse primary macrophages were isolated using a protocol described earlier (Titus et al., 1984) and kept in tissue culture plates for 48 h before further use.

Serum Tests

Blood samples were collected at the time of sacrifice of animals and serum was isolated after overnight separation at 4^oC and stored at -80^oC until further use. The following tests were performed: The lipid estimation kits from Merck were used employing enzymatic method for Cholesterol, Triglyceride and HDL and direct method for LDL. Serum glucose was measured using GODPOD method. Serum albumin was measured using BCG method.

Infection Score

Enumeration of organ parasites in liver was accomplished by microscopic evaluation of geimsa stained tissue imprints following the method of Stauber (Stauber, 1963). Leishman Donovan Unit (LDU) = No. of amastigotes/1000 nucleated cells x Weight of spleen or liver (in grams).

Tissue Homogenization and RNA and Protein Isolation

Liver samples stored at -80°C were thawed, dissected into small pieces and were resuspended either in RIPA buffer (150mM NaCl, 1% Triton X100, 0.5% SDS,0.25% Na-Deoxycholate, 50mM Tris-HCl, pH 8.0) for protein isolation or in Trizol (Invitrogen) for RNA isolation. The tissue was homogenized using a micropestle and centrifuged at 13,000xg for 15 min at 4°C. The clear supernatant was collected and either stored as protein lysate in -80°C or further processed to isolate RNA using the standard protocol.

Infection Progression in miR-122-Overexpressing Mouse Liver

miR-122 was overexpressed in mouse liver in an identical procedure described in experimental procedure section in the main text of this manuscript. After 2 doses of plasmid injection at an interval of 6 days, infection was delivered as usual. Subsequently, 3 more doses were administered and finally mice were sacrificed at 30 days post infection. Serum lipid profile, liver parasite burden and miR-122 expression levels were determined.

Technical Details of Real-Time Quantitative RT-PCR

Total RNA was extracted with Trizol reagent from snap frozen liver and RNA concentration was determined using a nanodrop. The miR-122, miR-16 and U6 levels were quantified with Taqman real-time RT-PCR miRNA detection assay kit (Applied Biosystem) following the manufacturer's instructions. 25 ng total RNA was used for miR-122 and U6 and 100 ng total RNA was used for miR-16 quantification in the reverse transcription (RT) reaction. The RT reaction was diluted twice in water and 1/4th of aliquots were subsequently used for RT-PCR amplification according to the manufacturer's instructions (Applied Biosystem). The RT reaction condition was: 16°C,30 min; 42°C,30 min;85°C,5 min,4°C,α. The PCR condition was: 95°C, 5 min; 95°C,15 sec; 60°C,1 min for 40 cycles.

mRNA of selected genes, pre-let7a and pre-miR122 quantification was done using standard SYBR assays kit (Invitrogen). The reverse transcription reaction was carried out with 1 µg of total RNA for mRNA and 400 ng for premiRNAs were used in Reverse Transcription reaction using M-MLV RT enzyme from Invitrogen according to manufacturer protocol. Firststrand cDNA was subsequently diluted twice in nuclease-free water

before addition to the RT-PCR reaction mixture. The Applied Biosystems 7500 real-time PCR instrument was used for amplification. The RT reaction condition was: 25°C,30 min;48°C,30 min;95°C,5 min,4°C,α. The PCR condition was: 95°C, 5 min; 95°C,15 sec;60°C,1 min for 40 cycles**.**

The primer sequences use for PCR amplification are as follows:

**GENE NAME**	**PRIMER SEQUENCE**	
	**FORWARD PRIMER**	**REVERSE PRIMER**
**HMGCR**	5`-ACTTTAATGGAAGCCAGTGG-3`	5`-CATCAAGGACAGCTCACC-3`
**ACAT2**	5`-TTTGCTCTATGCCTGCTTC-3`	5`-GTAGAACATCCTGTCTCC-3`
**SCD1**	5`- TTTCTACT ACATGACCAG CG-3`	5`- TCAGACATGTCCAGTTTTCC-3`
**CES1**	5`-ATGGTGTGGATACATGGAGG-3`	5`- ACAGAGACACTTTCACCTCC-3`
**CPT1B**	5`- AGACCTTCTTCAGCTCTGG-3`	5`-AGGTGAAAGGTATCAGTGCC-3`
**SGMS2**	5`- ATTCTTCGGCATCCTGAAGC-3`	5`-GCTACAAAATCTCCTTTCCG-3`
**premiR122**	5`-CCTTAGCAGAGCTGTGGAG-3`	5`-GCCTAGCAGTAGCTATTTAG-3`
**18S rRNA**	5`-TGACTCTAGATAACCTCGGG-3`	5`-GACTCATTCCAATTACAGGG-3’
**β-ACTIN**	5`- CCTCTATGCCAACACAGTGC-3`	5`- GTACTCCTGCTTGCTGATCC-3`

The result were normalised against 18S rRNA for mRNAs and against β-ACTIN for premiR-122.

Technical Details of Luciferase Reporter Assay

All transfections were performed using Lipofectamine 2000 (Invitrogen) following manufacturer’s instructions. For miRNA reporter expression assay, 25 ng of renilla (RL) reporter plasmids and 500 ng plasmid encoding firefly (FL) were transfected per well of a six-well plate. Other plasmids and small RNAs like pmiR-122 (500ng), let7a-mimic (100 pmol), miR-122 mimic (100 pmol), sinon-target (50 pmol), siDynamin 2 (50 pmol), pCI-FLAG-DICER1 (2 µg), pCI-HA-AGO2 (2 µg) and pCI-neo-GFP (2 µg) were

used at the indicated concentrations. Unless indicated otherwise, cells were split after 24 h. After 6 h of splitting, cells were either treated with the *L. donovani* parasite or, Leishmania exosomes or other chemicals for indicated duration before Huh7 cells were lysed in passive lysis buffer (Promega) and RL and FL activities were measured using Dual-Luciferase Assay Kit (Promega) measured on a VICTOR X3 Plate Reader systems (Perkin Elmer) and fold repression was calculated.

Plasmid Information

Description of plasmids pRL-Con and pFL were all published previously (Pillai et al., 2005) and were the kind gift from Witold Filipowicz. The pRL-3xBulge-miR-122 reporter contains three sites with a sequence AUACUAUUGUUAACACACACUCCA and cloned between XbaI and NotI site of pRL-Con. Upon base-pairing with miR-122, this sequence forms a duplex containing a bulge in its central region. Myc-AGO2, FLAG-HA-AGO2 plasmids were also described earlier (Kundu et al., 2012; Meister et al., 2004). GFP expression plasmids were generated by replacing RL encoding sequence of pRL-Con with GFP encoding fragment cloned in the NheI, XbaI site. A plasmid encoding precursor miR-122 (pmiR-122) was generated by inserting the PCR amplified fragment encoding the pre-miR-122 under a U6 promoter as described by Chang et al., 2004. Plasmids encoding pCI-neo, FLAG-DICER1 and NHA-DICER1-6xHis were also obtained from Witold Filipowicz laboratory.

Immunofluorescence

Huh7 cells or primary macrophage were plated on gelatine-coated cover slips at a confluency of approximately 20%. They were either transfected with GFP-tubulin or RFP-Actin or Endo-GFP encoding plasmids (Clontech) alone or along with si-nontarget or si-Dynamin 2 siRNAs. Subsequently cells were incubated with Ag83 strain of *L. donovani* promastigotes for 24 h or with Leishmanial exosome for 16 h in DMEM. Further, the cover slips were washed with PBS, the cells were fixed with 4% paraformaldehyde for 30 min in dark at room temperature before they were washed and stored in PBS at 4°C for further use. For immunofluorescence, cells were blocked and permeabilised in blocking buffer (with PBS containing 1%BSA, 1% goat serum and 0.1% Triton X-100 for 30min.) for 1 hour and incubated overnight with primary antibodies against gp63 and Dynamin 2 (antibody details listed below). Alexa Fluor® 488 conjugated

Phalloidin (Molecular Probes) was used at a dilution of 1:500 in PBS containing 1% BSA to stain actin in primary mouse macrophage. Signals were visualized after with Alexa Fluor ®488 or 594 tagged secondary antibodies while DAPI was used to stain host cell and parasite genomic DNA.

Image Capture and Postcapture Image Processing

Cells were imaged with an inverted Eclipse Ti Nikon microscope equipped with a Plan Apo VC 60X/1.40 oil objective and a Nikon Qi1MC camera for image capture except for the confocal imaging when the cells were observed with a 60X/N.A.1.42 Plan Apo N objective fitted on an ANDOR Spinning Disc Confocal Imaging System on an Olympus IX81 inverted microscope. Z stack images were captured with a IXON3 EMCCD camera. All images captured on Nikon Eclipse Ti microscope or ANDOR spinning disc microscope were analyzed and processed with Nikon NIS ELEMENT AR 3.1 software. Cropping was done using Adobe Photoshop CS4.

Purification of gp63 from *L. donovani* Lysate

gp63 was isolated following a published protocol (Bhowmick et al., 2008). Briefly, Soluble Leishmanial Antigens (SLA) were extracted from stationary phase *L. donovani* promastigotes harvested after the second passage as described earlier (Afrin et al., 2002) and almost 1×10^{10} promastigotes were suspended in ice-cold 5 mM Tris-HCl buffer (pH 7.6) containing 1X protease inhibitor cocktail (Roche). The suspension was vortexed six times for 2 min each with a 10 min interval on ice. The parasite suspension was then centrifuged at 2,310×g for 10 min, and the pellet was collected. The crude ghost-membrane pellet was resuspended in 10 ml of the same buffer and sonicated three times for 1 min each on ice. The suspension thus obtained was solubilized with lysis buffer containing 1% (wt/vol) octyl-β-D-glucopyranoside overnight at 4°C and was ultracentrifuged for 1 h at 100,000×g. The supernatant containing SLA was used for purification of gp63 as described previously (Russell and Wilhelm, 1986) with modifications. Two milligrams of SLA was applied to a Con A-Sepharose resin preequilibrated in the buffer used for SLA preparation containing 1% octyl-β-D-glucopyranoside and maintained at 4°C. Unbound protein was washed off the column with the same buffer, and bound material was eluted with 0.3 M α-methyl-D-mannoside in wash buffer. The gp63 enriched eluent was dialyzed against 5 mM Tris-HCl (pH 8.5) with 0.2% Zwittergent 3.12.

The dialyzed eluent (800 µg of protein) was loaded on a 1 ml DEAE-cellulose column that was preequilibrated in the same buffer used for dialysis. Under these conditions the anion exchanger readily bound gp63. Following thorough washing, the bound protein was eluted serially with 100, 300 and 500 mM NaCl in the same buffer. Eluted fractions were run on 10% SDS-PAGE and silver stained. gp63 enriched 300 mM NaCl eluent was used in subsequent assays.

Technical Details of In Vitro pre-miRNA Processing Assay

A 58 nucleotide synthetic pre-miRNA corresponding to the human pre-miR122 (**5'** UGGAGUGUGACAAUGGUGUUUGUGUCUAAACUAUCAAACGCCAUUAUCACACUAAAUA- **3'**) was 5' end labelled using [γ-^{32}P] ATP and T4 polynucleotide kinase. HEK293 cells stably expressing FLAG-HA-AGO2 were transiently transfected with NHA-DICER1 expressing plasmid. 5 x 10^6 cells were harvested 48 hours after transfection and lysed in a 200 µl buffer containing 10mM Tris pH 7.5, 200mM KCl, 5mM $MgCl_2$, 1mM DTT, 1X EDTA-free Protease Inhibitor, 1% Triton X-100, followed by sonication. The cell lysate was clarified by spinning twice at 16000xg for 10min. The lysate was incubated with 1 U of purified gp63 [1 U of gp63 defined as the amount of gp63 that cleaves 50% DICER1 in 30 min at 37ºC in cell lysate expressing NHA-DICER1 in an *in vitro* reaction] for 30 min at 37ºC followed by immunoprecipitation with Anti-FLAG M2 affinity gel (SIGMA) overnight at 4ºC. After washing thrice with IP Buffer (20mM Tris-HCl, pH 7.5, 150mM KCl, 5mM $MgCl_2$, 1mM DTT, 1X Protease Inhibitor), two-third of the beads was subjected to western blotting and the remaining was used for pre-miR122 processing assay with 10 nM pre-miR122 in a buffer containing 20mM Tris pH 7.5, 200mM KCl, 5mM $MgCl_2$, 1mM DTT, 5% glycerol at 37ºC for 60 min. RNA was isolated from the reactions and the products analysed on a 12% denaturing (8M Urea) polyacrylamide gel. Phosphoimaging of the dried gels were performed in Cyclone Plus Storage Phosphor System (Perkin Elmer).

BrdU Incorporation and Cell Growth Assay

Huh7 cells were plated in 96 well plate at 2.5x 10^4 per well. Cells were treated with 2.5x 10^5 number of *L. donovani* promastigote or were left untreated. After 20 h incubation, BrdU solution was added to the cells and further incubated for another 3-4 h. The level of BrdU incorporation was measured according to

manufacturer’s protocol (Millipore) and the absorbance was measured at 450 nm using ELISA plate reader (DTX 800 multimode detector: Beckman Coulter).

Histochemical Analysis of Parasite-Infected Mouse Liver

To score hepatic tissue response in mice, liver sections from normal, 15, 30 and 60 days infected and DICER1 overexpressed mice were assessed microscopically after staining with Haematoxyline and Eosin as previously described by others (McElrath et al., 1988; Murray, 2001). Briefly, portions of liver tissue were first fixed in 10% formalin, and tissue sections were stained after standard processing like paraffin embedding, histological sectioning and mounting on glass slides. Liver sections were examined by Nikon Eclipse E200 using 40X magnification. Granuloma formation was visualized and quantified in infected liver sections. Diameter of granuloma was calculated and number of granuloma per field counted.

For immunofluorescence detection of gp63 in mouse liver, 30 days infected liver cryosections were prepared (20 micron thickness) on polylysine coated glass slides in a cryotome (Thermo Electron Corporation, SHANDON CRYOTOME E) and fixed in 100% ethanol overnight. For immunostaining slides were hydrated in PBS for 4 h at room temperature. Sections were permeabilized using blocking and permeabilization buffer containing 0.5% Triton X100 for 1 h at room temperature. Subsequently, the slides were washed with PBS (5min x3) and treated with anti gp63 antibody (1:500 dilution) at 4°C overnight in a humid chamber. The sections were further washed with PBS (5min x3) and stained using anti-mouse-IgG-Alexa-594 (1:500 dilution) and Plalloidin-Alexa-488 (to stain F-Actin for visualizing the cellular boundaries) (1:500 dilution) at room temperature for 1 h. The slides were again washed with PBS (5min x3). Lastly coverslip was mounted using Vectasheild-DAPI (for nuclear staining). Finally images were captured on ANDOR Spinning Disc Confocal Imaging System installed on an Olympus IX81 inverted microscope.

Liposome Preparation and gp63 Trapping

gp63 entrapment in liposome was done using a previously described procedure (Bhowmick et al., 2008) with slight modifications. Briefly, 24 mg Phosphatidylcholine (Avanti Polar Lipids) (100mg/ml), 17.4 mg Cholesterol (Sigma) (100mg/ml) (1:1.5::Cholesterol to PC molar ratio) (Banerjee et al., 2009) and 6 mg n-

octyl-β-D-glucopyranoside (Sigma) (~1.5 mg/ 10 mg lipid) was dissolved in chloroform. A uniform monolayer was coated in a glass test tube and residual removal of the solvent was done. The lipid layer was solubilized in PBS containing either 400 µg purified gp63 (~100 µg /10 mg lipid) or in absence of gp63. The suspension was vortexed (prior to addition of the protein) and sonicated in a bath sonicator for 15 min on ice water. The sonicated liposomal suspension was dialysed against PBS overnight at 4°C. The dialysed liposomes were centrifuged at 1,00,000 x g at 4°C for 1 hour to remove unincorporated protein and lipid particles. It was further resuspended in either saline (for animal injection) or DMEM medium (for cell culture use). Percent entrapment was determined by protein estimation method. 80% gp63 protein entrapment was observed.

NBD-Liposomes were prepared using NBD-cholesterol (Avanti Polar Lipid), the fluorescent analogue of cholesterol used to study cellular uptake (Portioli Silva et al., 2004). Liposomes were prepared as usual with 1% NBD-cholesterol of total cholesterol used.

Live Animal Imaging

Live Animal Imaging was done according to Jash.S et al (Jash et al., 2012). Briefly, 2 months old mice were injected with 100 µl of NBD-liposomal suspension through cardiac route and imaged under ketamine or isofluorane anesthesia in a Xenogen IVIS Spectrum Imaging chamber. Fluorescence imaging was performed under epi-illumination with excitation/emission filter setting at 470/510 nm. Concentration contour plots were obtained using Living Image 3.0 software. Region of Interest (ROI) were visible.

Polysome Analysis

For polysome analysis, around $1x10^7$ Huh7 cells were lysed in a buffer containing 10 mM HEPES pH 8.0, 25 mM KCl, 5 mM $MgCl_2$, 1 mM DTT, 5mM vanadyl ribonucleoside complex, 1% Triton X-100, 1% sodium deoxycholate, and 1X EDTA-free protease inhibitor cocktail (Roche) supplemented with CHX (100 µg/ml; Calbiochem). Gradient analysis was performed as described previously (Bhattacharyya et al., 2006; Pillai et al., 2005). The absorbance profile was obtained by using an ISCO UA-6 absorbance monitor and fractions were collected on an ISCO gradient fractionator.

Statistical Analysis

All experiments were done thrice and only representative data are presented, interassay variation being within 5-10%. All micrographs were processed with Adobe Photoshop Elements7. All graphs and statistical analyses were generated in GraphPad Prism 5.00 (GraphPad, San Diego,CA). Nonparametric unpaired t test was used for analysis. We considered P values < 0.05 to be statistically significant. P values < 0.001 were considered extremely significant (***), ranging between 0.001 to 0.01 as very significant (**), ranging between 0.01 to 0.05 as significant (*) > 0.05 were not significant (ns). Error bars indicate mean ± S.D.

Antibody used for Western Blots and Immunofluorescence

**Name of Antigen**	**Raised in**	**Source**	**Dilutions used for Western Blot / Immunofluorescence**
HMG-CoA-receptor	Rabbit	Santa Cruz Biotechnology	1:1000
ACAT 2	Rabbit	Novus Biologicals	1:500
Dynamin 2	Rabbit	Sigma-Aldrich	1:1000 (Western Blot)<br>1:500 (Immunofluorescence)
AGO 2	Mouse	Novus Biologicals	1:500
RCK/p54	Rabbit	Bethyl	1:5000
DICER1	Rabbit	Bethyl	1:2500
β-ACTIN	Mouse (HRP conjugated)	Sigma	1:10000
HA	Rat	Roche	1:1000

DGCR 8	Rabbit	Bethyl	1:5000
gp63	Mouse	LS Biosciences	1:500 and 1:10 for antibody blocking
gp63	Rabbit (polyclonal)	Antisera raised in rabbit	1:2500 and 1:10 for antibody blocking
GFP	Mouse	Roche	1:10 for antibody blocking
GRP78	Rabbit	Sigma	1:10 for antibody blocking

Supplemental References

Afrin, F., Rajesh, R., Anam, K., Gopinath, M., Pal, S., and Ali, N. (2002). Characterization of Leishmania donovani antigens encapsulated in liposomes that induce protective immunity in BALB/c mice. Infection and immunity *70*, 6697-6706.

Bhattacharyya, S.N., Habermacher, R., Martine, U., Closs, E.I., and Filipowicz, W. (2006). Relief of microRNA-mediated translational repression in human cells subjected to stress. Cell *125*, 1111-1124.

Jash, S., Chowdhury, T., and Adhya, S. (2012). Modulation of mitochondrial respiratory capacity by carrier-mediated transfer of RNA in vivo. Mitochondrion *12*, 262-270.

Kundu, P., Fabian, M.R., Sonenberg, N., Bhattacharyya, S.N., and Filipowicz, W. (2012). HuR protein attenuates miRNA-mediated repression by promoting miRISC dissociation from the target RNA. Nucleic Acids Res.

McElrath, M.J., Murray, H.W., and Cohn, Z.A. (1988). The dynamics of granuloma formation in experimental visceral leishmaniasis. The Journal of experimental medicine *167*, 1927-1937.

Meister, G., Landthaler, M., Patkaniowska, A., Dorsett, Y., Teng, G., and Tuschl, T. (2004). Human Argonaute2 mediates RNA cleavage targeted by miRNAs and siRNAs. Mol Cell *15*, 185-197.

Murray, H.W. (2001). Tissue granuloma structure-function in experimental visceral leishmaniasis. International journal of experimental pathology *82*, 249-267.

Pillai, R.S., Bhattacharyya, S.N., Artus, C.G., Zoller, T., Cougot, N., Basyuk, E., Bertrand, E., and Filipowicz, W. (2005). Inhibition of translational initiation by Let-7 MicroRNA in human cells. Science *309*, 1573-1576.

Portioli Silva, E.P., Peres, C.M., Roberto Mendonca, J., and Curi, R. (2004). NBD-cholesterol incorporation by rat macrophages and lymphocytes: a process dependent on the activation state of the cells. Cell biochemistry and function *22*, 23-28.

Russell, D.G., and Wilhelm, H. (1986). The involvement of the major surface glycoprotein (gp63) of Leishmania promastigotes in attachment to macrophages. J Immunol *136*, 2613-2620.

Stauber, L.A. (1963). Immunity to Leishmania. Ann N Y Acad Sci *113*, 409-417.

Titus, R.G., Kelso, A., and Louis, J.A. (1984). Intracellular destruction of Leishmania tropica by macrophages activated with macrophage activating factor/interferon. Clin Exp Immunol *55*, 157-165.
