# Supplementary figures and images for: Leishmania donovani Targets Dicer1 to Downregulate miR-122, Lower Serum Cholesterol, and Facilitate Murine Liver Infection
Source: Cell Host Microbe. 2013 Mar 13;13(3):277–88. doi: 10.1016/j.chom.2013.02.005 (PMC3605572; doi:10.1016/j.chom.2013.02.005)

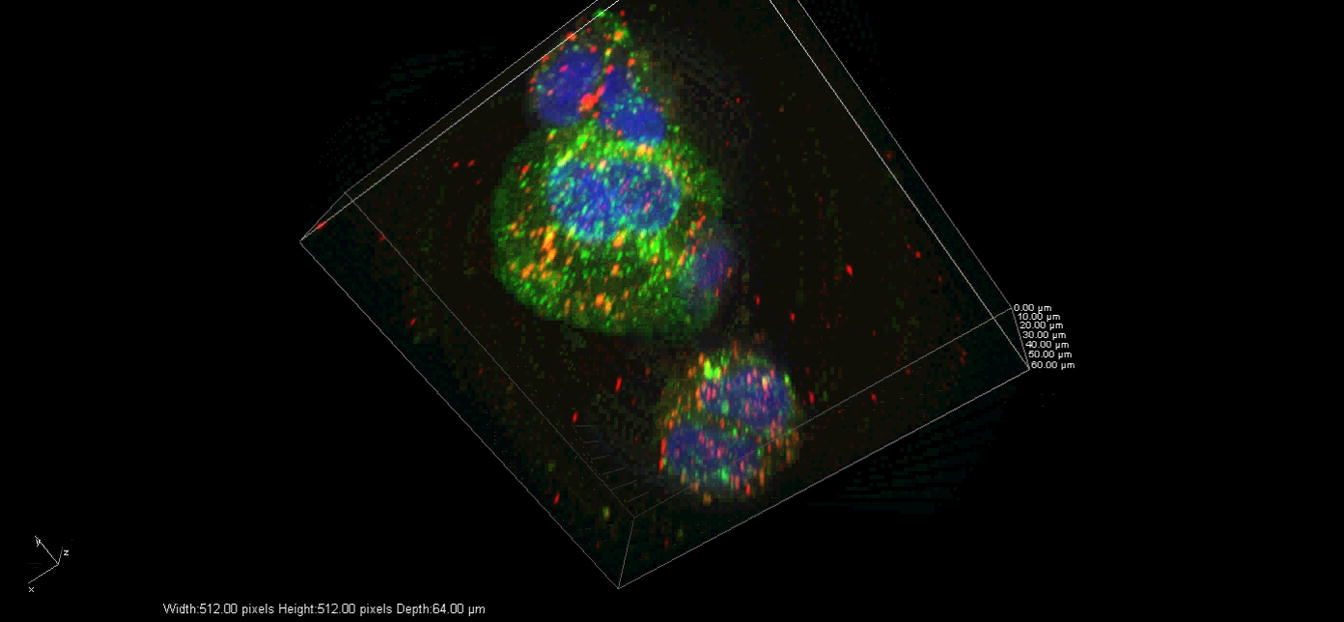

Supplement: Movie S1. Proximal Association or Colocalization of Internalized gp63 with GFP-Tagged Endosomes in L. donovani Exosome-Treated Huh7 Cells, Related to Figure 4 — z-stacked merged images of Endo-GFP-expressing Huh7 cells treated with L. donovani exosomes and stained for gp63 were captured with an ANDOR Spinning Disc Confocal microscope and processed with Nikon NIS Element AR 3.2 software to get the 3D view of the cells. The 3D rotational views were captured at a rate of 30 frames/sec to create the movie. Endo-GFP is in green and gp63 is in red. DAPI was used to stain the nucleus. [file mmc3.jpg]
